# Feasibility and preliminary effects of exercise interventions on plasma biomarkers of Alzheimer’s disease in the FIT-AD trial: a randomized pilot study in older adults with Alzheimer’s dementia

**DOI:** 10.1186/s40814-022-01200-2

**Published:** 2022-12-02

**Authors:** Fang Yu, Seung Yong Han, Dereck Salisbury, Jeremy J. Pruzin, Yonas Geda, Richard J. Caselli, Danni Li

**Affiliations:** 1 Edson College of Nursing and Health Innovation, Arizona State University, Phoenix, AZ USA; 2 Adult and Gerontological Health Cooperative, School of Nursing, University of Minnesota, Minneapolis, MN USA; 3 Department of Neurology, Banner Alzheimer’s Institute, Phoenix, AZ USA; 4 Department of Neurology, and Franke Barrow Global Neuroscience Education Center, Barrow Neurological Institute, Phoenix, AZ USA; 5 Department of Neurology, Mayo Clinic Arizona, Phoenix, AZ USA; 6 Department of Lab Medicine and Pathology, University of Minnesota, Minneapolis, MN USA

**Keywords:** Alzheimer’s disease, Exercise, Feasibility, Pilot, Biomarkers

## Abstract

**Background:**

Alzheimer’s disease (AD) biomarkers have provided a unique opportunity to understand AD pathogenesis and monitor treatment responses. However, exercise trials show mixed effects on imagining and cerebrospinal fluid biomarkers of AD. The feasibility and effects of exercise on plasma biomarkers remain unknown. The primary objective of this study was to examine the feasibility of recruitment, retention, and blood sample collection in community-dwelling older adults with mild-to-moderate AD dementia. Secondarily, it estimated the preliminary effects of 6-month aerobic and stretching exercise on plasma amyloid-β_42_ and Aβ_40_ (Aβ_42/40_) ratio, phosphorylated tau (p-tau) 181, and total tau (t-tau).

**Methods:**

This pilot study was implemented in year 2 of the 2-parallel group FIT-AD trial that randomized 96 participants on a 2:1 allocation ratio to moderate-intensity cycling or low-intensity stretching for 20–50 min, 3 times/week for 6 months with 6-month follow-up. Investigators (except for the statistician) and data collectors were blinded to group assignment. Fasting blood samples were collected from 26 participants at baseline and 3 and 6 months. Plasma Aβ_42_, Aβ_40_, p-tau181, and t-tau were measured using Simoa™ assays. Data were analyzed using intention-to-treat, Cohen’s d, and linear mixed models.

**Resultss:**

The sample averaged 77.6±6.99 years old and 15.4±3.00 years of education with 65% being male and 96.2% being apolipoprotein epsilon 4 gene carriers. The recruitment rate was 76.5%. The retention rate was 100% at 3 months and 96.2% at 6 months. The rate of blood collection was 88.5% at 3 months and 96.2% at 6 months. Means (standard deviation) of within-group 6-month difference in the stretching and cycling group were 0.001 (0.012) and −0.001 (0.010) for Aβ_42/40_ ratio, 0.609 (1.417) pg/mL and 0.101(1.579) pg/mL for p-tau181, and −0.020 (0.279) pg/mL and −0.075 (0.215) pg/mL for t-tau. Effect sizes for within-group 6-month difference were observed for p-tau181 in stretching (*d*=0.43 [−0.33, 1.19]) and t-tau in cycling (−0.35 [−0.87, 0.17]).

**Conclusions:**

Blood collections with fasting were well received by participants and feasible with high recruitment and retention rates. Plasma biomarkers of AD may be modifiable by exercise intervention. Important design considerations are provided for future Phase III trials.

**Trials registration:**

ClinicalTrials.gov Identifier: NCT01954550 and posted on October 1, 2013

**Supplementary Information:**

The online version contains supplementary material available at 10.1186/s40814-022-01200-2.

## Key messages regarding feasibility

- What uncertainties existed regarding the feasibility?
  Plasma biomarkers are increasingly validated to closely correlate with AD pathologies measured in the brain; however, exercise trials show mixed effects on imagining and cerebrospinal fluid biomarkers of AD. The feasibility and effects of exercise on plasma biomarkers remain unknown.
- What are the key feasibility findings?
  Blood collections were well received by participants, including the need to fast for 8 hours prior to collections. The recruitment rate was 76.5% with a passive recruitment strategy. The retention rate was 100% at 3 months and 96.2% at 6 months, losing only one participant due to non-study-related death. The rate of blood collection was 88.5% at 3 months and 96.2% at 6 months. Plasma p-tau181 may be modifiable by exercise.
- What are the implications of the feasibility findings for the design of the main study?
  Blood biomarkers have the potential to significantly broaden AD research because they could be easily integrated into intervention studies that are often limited by resources to use the more established but invasive and expensive ways to measure biomarkers. Our findings indicate that future RCTs could improve recruitment success with active recruitment, even at minimal staff effort. The blood collection rate can be increased by using a wider data collection window than the 1-week used in our study, providing options for blood collections at home or a local laboratory, and work with participants and families to overcome perceived barriers. Plasma p-tau181 may be modifiable by exercise, which needs to be tested in future RCTs.

## Background

Alzheimer’s disease (AD) affects more than six million Americans currently and is projected to affect 14 million at a cost of $1.2 trillion in 2050 [1]. Despite its tremendous impact, AD cannot yet be prevented or cured, even with recent medications that reduced amyloid-beta (Aβ) burden, a hallmark AD pathology, in the brain [2]. Physical inactivity contributes to 17% of dementia cases [3]. Compared to no or low physical activity, high physical activity is linked to about 38% risk reduction for AD [4] and moderates the association of AD pathology and cognitive decline in cognitively normal older adults [5]. Meta-analyses of randomized controlled trials (RCTs) found that aerobic exercise interventions produced modest to moderate cognitive gains in older adults with mild cognitive impairment or AD dementia [6]. Extensive evidence from animal research strongly supports that physical activity, particularly aerobic exercise, modifies the AT(N) biomarkers of AD [7]. AT(N) refers to Aβ, hyperphosphorylated Tau, and neurodegeneration. Recent technological breakthrough has made it possible to measure AT(N) in the blood [8, 9].

In AD, Aβ_42_ aggregates in the brain parenchyma, resulting in a 50% reduction in its concentration in the cerebrospinal fluid (CSF) [10] and 14–20% reduction in the plasma [11–15]. Aβ_42/40_ ratio, the concentration of Aβ_42_ divided by the concentration of Aβ_40_, normalizes inter-individual differences in Aβ production [8]. CSF Aβ_42/40_ ratio shows almost 100% concordance to positron emission tomography (PET) amyloid load [16]. Later, low plasma Aβ_42/40_ ratio was found to reflect PET amyloid load and CSF Aβ_42/40_ ratio [11–15, 17, 18]. In addition to Aβ, plasma tau hyperphosphorylated at threonine 181 (p-tau181) and 217 (p-tau217) can accurately detect PET amyloid and tau pathology, predict future progression to AD dementia, and differentiate AD from non-AD neurodegeneration [19–24]. Plasma p-tau181 correlates well to CSF p-tau181, PET amyloid load, and PET tau load [25, 26]. Changes in plasma p-tau181 were reported to become significant prior to PET amyloid abnormality but after abnormal CSF and plasma Aβ_42_ changes [26], suggesting that plasma p-tau181 detects Aβ-induced tau pathology years earlier than tau PET [27]. On the other hand, t-tau reflects neuronal injury with CSF and plasma t-tau performing similarly, especially in acute brain injuries, and may reflect Aβ-induced tau secretion in AD [8].

AT(N) biomarkers provide an emerging opportunity to study treatment responses in AD. In cognitively normal older adults, 52-week aerobic exercise did not affect PET amyloid load [28]. In older adults with mild cognitive impairment, a 6-month aerobic exercise reduced plasma Aβ_42_ by ~6% while stretching increased it by 24% [29]. In Danish older adults with mild-to-moderate AD dementia, a 16-week aerobic exercise did not change CSF Aβ_42_ [30], CSF tau [31], and PET amyloid [32]. In the USA, a 6-month aerobic exercise showed a trend of reduced hippocampal atrophy and significantly decreased white matter hyperintensity in comparison to stretching exercise in older adults with mild-to-moderate AD dementia [33]. However, no studies have examined the feasibility of blood biomarkers in older adults with Alzheimer’s dementia or the effects of aerobic exercise on plasma AD biomarkers in older adults with AD dementia.

This study was the blood ancillary study of the FIT-AD trial and a randomized pilot study according to the conceptual framework by Eldridge and colleagues [34] because the FIT-AD trial was a phase II pilot trial to test all non-blood aspects of the future Phase III RCT and this study piloted the blood component. The primary objective of this study was to examine the feasibility of recruitment, retention, and blood collection in community-dwelling older adults with mild-to-moderate Alzheimer's dementia. Secondarily, it estimated the preliminary effects of a 6-month aerobic and stretching exercise on plasma Aβ_42/40_ ratio, p-tau181, and t-tau. We predicted that recruitment, retention, and blood collection rates would be high (>80%) and preliminary effects on AD plasma biomarkers would differ between aerobic and stretching exercises.

## Methods

### Design

The FIT-AD trial examined the effects of aerobic exercise on cognition and hippocampal volume. Ninety-six community-dwelling older adults with mild-to-moderate Alzheimer's dementia were randomized on a 2:1 allocation ratio to moderate-intensity cycling or low-intensity stretching for 20–50 min, 3 times a week for 6 months within 3 age strata (age 66–75, 76–85, and 85+ years) using random permuted blocks of 3 and 6 generated by the statistician and followed them for an additional 6 months. The randomization schedule was concealed in opaque envelopes from all investigators (except for the statistician) and data collectors. The details of the FIT-AD trial protocol and its main cognitive and imaging biomarker findings were published previously [33, 35, 36]. Procedures involving experiments on human subjects were done in accordance with the ethical standards of the Committee on Human Experimentation of the institution in which the experiments were done or in accord with the Helsinki Declaration of 1975.

**Fig. 1:**
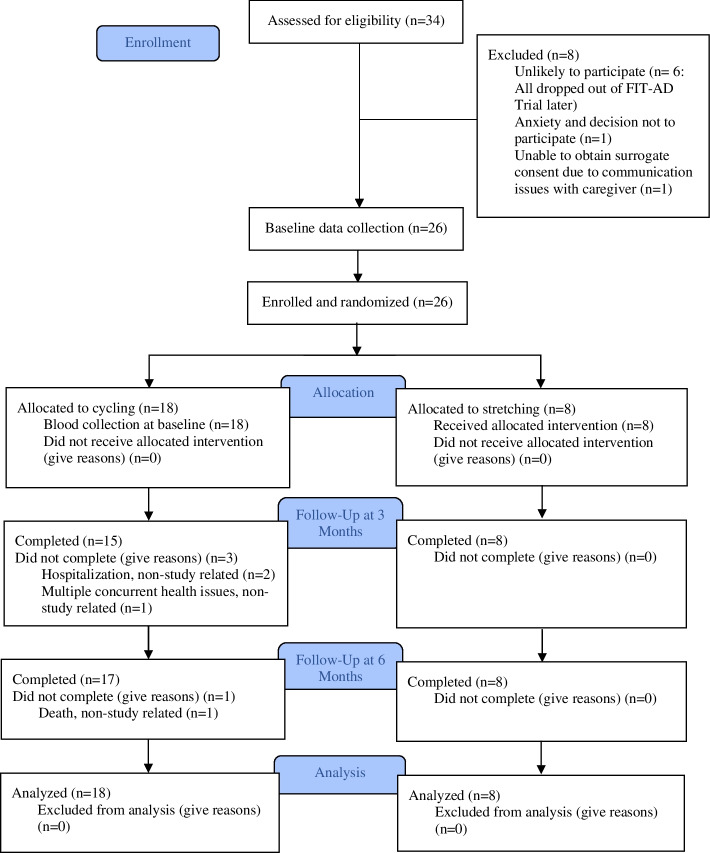
CONSORT diagram for blood collections

The FIT-AD blood study was launched in year 2 of the FIT-AD Trial (June 2015) after 30 participants were already enrolled in the FIT-AD trial, leaving us with a large pool of potential participants to recruit from. Given the target enrollment goal of 25, we took a very passive recruitment approach. Once potential FIT-AD participants passed the FIT-AD Trial screening, the study staff emailed the study recruitment material to them or their caregivers and asked them to contact us if interested. In total, we emailed 44 consecutive potential participants of the FIT-AD trial and 10 did not respond. For those who responded (*n*=34), we screened out 8 who were unlikely to participate due to their over-committed schedules, over-burdened caregivers, or long travel distance for blood collections. We stopped recruitment once we reached our enrollment goal in June 2017. As a result, 26 participants were consented during FIT-AD baseline data collection and enrolled in the blood study (Fig. 1). Upon completing FIT-AD baseline data collection, participants were formally enrolled in the FIT-AD trial and randomized to start their assigned exercise within 2 weeks. Blood collection occurred within this 2-week window and then at 3 months and 6 months by phlebotomists who were blinded to the study aims and randomization. The CONSORT checklist is provided as an additional file. This study was approved by the University of Minnesota Institutional Review Board (IRB: #1508M77566).

### Sample and sample size

Inclusion criteria for the FIT-AD trial were community-dwelling older adults 66+ years old with mild-to-moderate AD dementia; AD dementia was diagnosed clinically and verified by participants’ primary care providers and by the investigators using 2011 diagnostic criteria [37]; verified exercise and magnetic resonance imaging (MRI) safety; English-speaking; Mini-Mental State Examination (MMSE) scores 15–26; Clinical Dementia Rating (CDR) scores of 0.5–2; and stable on medications to treat AD >1 month if prescribed. Participants were excluded if their resting heart rate was >100 or <50 beats per minute; had neurological, psychiatric, or substance disorders in the past 5 years that may explain cognitive impairment; and abnormal signs, symptoms, and conditions uncovered during screening.

The target sample size for this pilot study was 25 based on the recommended sample size for pilot studies [38–40]. To be eligible for this study, participants enrolled in the FIT-AD trial had to agree to donate 20-mL blood at each collection (60mL total) and fast for at least 8 h (no food or drink other than water and prescribed medications) before blood collection. The final sample sizes for stretching and cycling groups were 8 and 18 at baseline, 8 and 15 at 3 months, and 8 and 17 at 6 months, respectively.

### Setting

The study was carried out at the University of Minnesota. Transportation was provided by the study staff to bring participants to the Clinical and Translational Science Institute for blood collection. Experienced phlebotomists drew blood. After each blood draw, blood specimens were stored immediately on wet ice and transported by the study staff to author DL’s lab where a lab technician processed and aliquoted the specimens according to an established protocol. Plasma ethylenediaminetetraacetic acid (EDTA) tubes were mixed and centrifuged in 4°C using a temperature-controlled centrifuge with a Swing out Rotor at 1439 g for 15 min. Immediately after completion, the tubes were removed from the centrifuge and plasma samples were aliquoted and stored in a −80°C freezer [41].

### Intervention

The intervention was cycling on recumbent stationary cycles for 20–50 min at 50–75% of heart rate reserve (HRR) or 9–15 on the 6–20 Borg Ratings of Perceived Exertion (RPE), three times a week for 6 months (72 sessions). The dose of cycling was increased over time from 50–55% of HRR or RPE 9–11 for 20–30 min to 55–60% of HRR or RPE 10–12. Dose increases were alternated between 5% of HRR (1-point on RPE) intensity increase or 5-min session duration prolongation until the target dose of 70–75% HRR (or RPE 12–14) and 50 min was achieved.

The control exercise was seated movements and static stretches at low intensity (<20% of HRR or <RPE 9) and matched to the cycling group by session duration and frequency and the total number of sessions. To match the cycling session duration, the number of repetitions and durations for each movement and stretch was gradually increased.

Each session lasted 40–60 min, including a 5-min warm-up and a 5-min cool-down. Participant to interventionist ratio never exceeded 3:1. During each session, the interventionist monitored HR with a Polar HR monitor, RPE, talk test (ability to talk a sentence without losing breath), and over-exertion signs and symptoms.

### Variables and their measures

#### Primary feasibility outcomes

The primary feasibility outcomes included the recruitment rate, retention rate, and blood collection rate. The recruitment rate was calculated as the percent of potential participants who were enrolled among those who responded to our recruitment. The retention rate was defined as the percentage of participants who remained in the study among those who were enrolled at baseline. The rate of blood collection referred to the percentage of participants whose blood collection and pre-analytical processing were performed according to the established guidelines [42] among enrolled participants.

#### Secondary patient-centered outcomes

Plasma Ab_42/40_ ratio, p-tau181, and t-tau were measured using commercially available Simoa™ Human Neurology 3-plex A Kit (Quanterix) according to the manufacturer’s instructions. Each sample was run in duplicate. The total coefficient of variation percentage (CV %) for Ab_42_, Ab_40,_ and t-tau, determined using triplicate measurement of quality control (QC) samples across two plates, were 8%, 7%, and 10%, respectively. Plasma p-tau181 was measured using commercially available Simoa™ P-tau181(V2) kits (Quanterix) according to the manufacturer’s instructions. Each sample was run in duplicate. Total CV% for p-tau181 determined using triplicate measurement of 2 levels of QC samples across two plates were 7% and 13%, respectively.

#### Potential covariates

Potential covariates included demographics (age, sex, education, and marital status), which were assessed using a questionnaire and baseline cognition as measured by the MMSE. APOE genotype was determined by extracting DNA from packed cells stored at −80°C using Puregene® reagents (Qiagen, Germantown, MD) as e2/e2, e2/e3, e3/e3, e3/e4, e2/e4, or e4/e4 using Taqman® SNP Genotyping assays for rs429358 and rs7412 (Life Technologies, Carlsbad, CA).

### Statistical analyses

Descriptive statistics with means/standard deviations (SD) and frequencies/% were used to summarize the demographic and clinical characteristics of the participants at baseline (*N*=26). Statistical assumptions were checked prior to analysis. Independent samples *t* test for continuous measures (age and MMSE) and chi-square test for categorical measures (gender, APOE genotype, education, marital status, and race) were used to test differences in baseline characteristics between the cycling and stretching groups. Shapiro-Wilk and Shapiro-Francia tests showed the violation of the normality assumption for MMSE, but the *t* test results held after transformation. The observed levels of within-group differences in plasma Ab_42/40_ ratio, t-tau, and p-tau181 were summarized with means and SD.

Primary feasibility outcomes were determined based on their operational definitions in the Methods section. For secondary patient-centered outcomes, a linear mixed model (multilevel model) was used with time (level 1) nested within participants (level 2) to test the interaction between time (baseline, 3, and 6 months) and intervention group (stretching and cycling) for each outcome. A linear mixed model is appropriate to handle missing by using all available data without imputation for missing data and longitudinal data in which the correlations between observations within each participant are expected. In all linear mixed models, random variation in the initial status was allowed (hence the random-intercept model with the identity covariance structure), and the independent residual error structure within level 1 (time) was assumed. The variance components of each model assess the amount of outcome variability at levels 1 and 2 left after fitting the model. The level 2 variance component summarizes the between-participant variability in initial status after controlling for time and group as well as the covariates. A shape of trajectory was not assumed for any outcome so that time was treated as categorical in all models to compare baseline with 3 and 6 months.

To compute the effect sizes for within-group differences, pooled SD was used [43]. Bootstrapped 95% confidence intervals of the effect sizes were obtained after 1010 replications to ensure at least 1000 successful replications. Stata version 17.0 was used for all analyses which followed the intention-to-treat principle.

## Results

The participants were on average 77.6 years old (SD=6.99), male (65.4%), married (73.1%), and non-Hispanic White (92.3%) with 15.4 (SD=3.00) years of education and MMSE scores of 21.6 (SD=3.32). Most of the sample (96.2%) carried at least one copy of APOE e4. Participants attended 84.8% (SD=15.4%) of their assigned sessions. There were no significant differences in any of these variables at baseline between the stretching and cycling groups (Table 1).

**Table 1:** Characteristics of the study sample

	Overall		Stretching		Cycling	
	*N*	Mean±SD (range) or %^a^	*N*	Mean±SD (range) or %^a^	*N*	Mean±SD (range) or %^a^
Age in years	26	77.6±6.99 (66–93)	8	79.3±−5.5 (72.0–86)	18	76.8±7.6 (66–93)
MMSE	26	21.6±3.32 (15–26)	8	22.3±2.3 (19.0–26)	18	21.3±3.7 (15–26)
Education in years	26	15.4±3.00 (9–23)	8	14.5±2.5 (13–20.0)	18	15.9±3.2 (9–23)
Gender	26	100.0%	8	100.0%	18	100.0%
Male	17	65.4%	6	75.0%	11	61.1%
Female	9	35.6%	2	25.0%	7	38.9%
APOE genotype	26	100.0%	8	100.0%	18	100.0%
ε2/ε3	1	3.8%	0	0.0%	1	5.6%
ε3/ε4	11	42.3%	5	62.5%	6	33.3%
ε2/ε4	7	26.9%	1	12.5%	6	33.3%
ε4/ε4	7	26.9%	2	25.0%	5	27.8%
Marital Status	26	100.0%	8	100.0%	18	100.0%
Married	19	73.1%	7	87.5%	12	66.7%
Not married	7	26.9%	1	12.5%	6	33.3%
Race	26	100.0%	8	100.0%	18	100.0%
White	24	92.3%	7	87.5%	17	94.4%
Non-White	2	7.7%	1	12.5%	1	5.6%
Exercise adherence	26	84.8%±15.4%	8	92.9%±6.3%	18	81.3%±16.9%

The recruitment rate was 76.5%. The retention rate was 100% at 3 months and 96.2% at 6 months (1 lost to follow-up due to non-study-related death). The rate of the blood collection was 88.5% at 3 months (2 missed due to non-study-related hospitalization and 1 missed due to non-study-related multiple concurrent health issues [e.g., bell’s palsy, back surgery, sepsis]) and 96.2% at 6 months (1 missed due to non-study-related death).

**Fig. 2:**
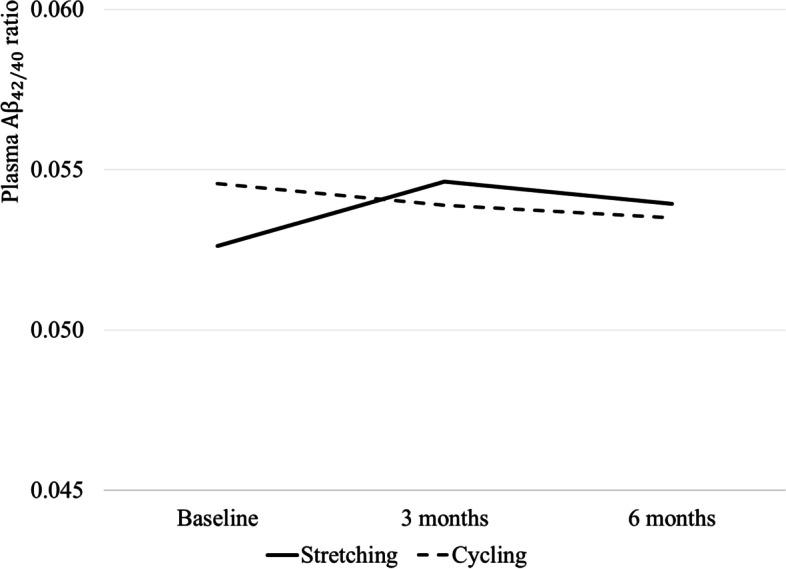
Observed levels of plasma ratio at baseline, 3 months, and 6 months by groups. Note: The sample sizes for the stretching and the cycling groups are 8 and 18, 8 and 15, and 8 and 17 at baseline, 3 months, and 6 months, respectively

**Fig. 3:**
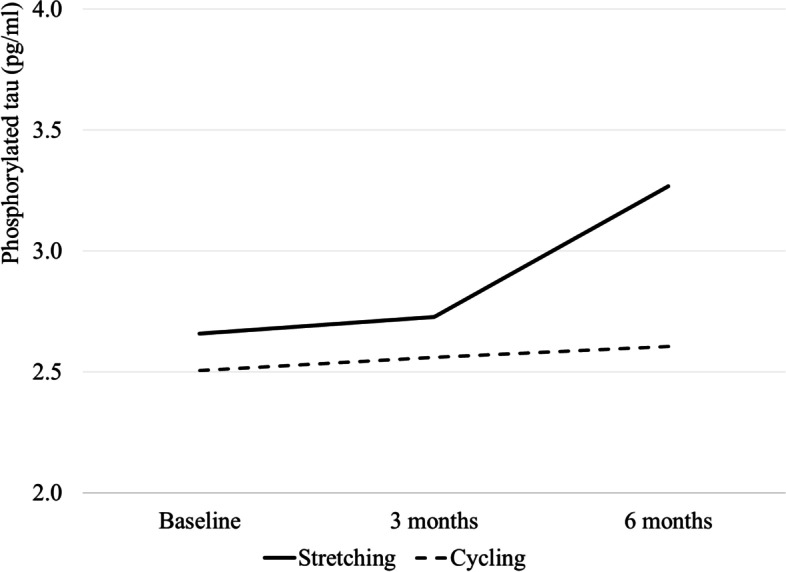
Observed levels of p-tau181 at baseline, 3 months, and 6 months by groups. Note: The sample sizes for the stretching and the cycling groups are 7 and 15, 8 and 15, and 8 and 16 at baseline, 3 months, and 6 months, respectively

**Fig. 4:**
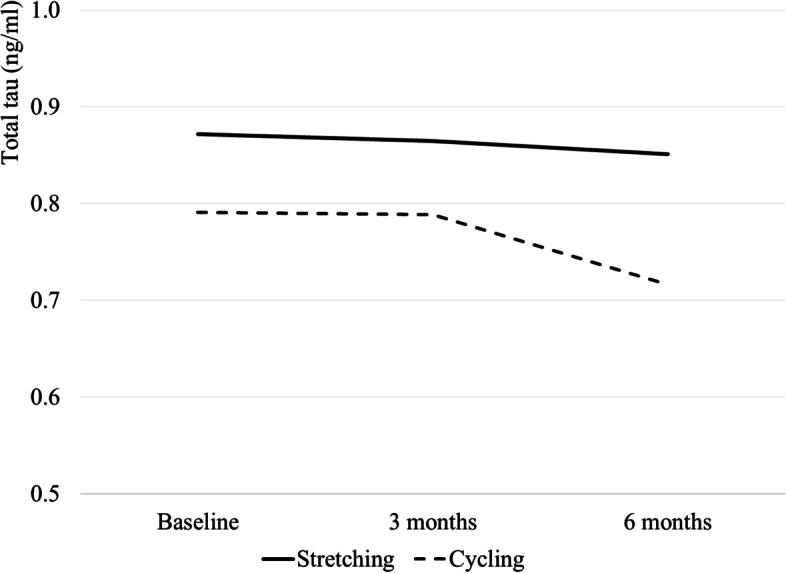
Observed levels of t-tau at baseline, 3 months, and 6 months by groups

Table 2 provides summary statistics (mean and SD) of plasma AD biomarkers by the group at baseline, 3 months, and 6 months. Figures 2, 3, and 4 graph plasma Aβ_42/40_ ratio, p-tau 181, and t-tau at baseline and over time at 3 and 6 months by group, respectively. Table 3 describes within-group differences in plasma AD biomarkers using baseline as reference (3 or 6 month - baseline) and effect sizes. Means (standard deviation) of within-group 6-month difference in the stretching group and cycling group were 0.001 (0.012) and −0.001(0.010) for plasma Aβ_42/40_ ratio, 0.609 (1.417) pg/mL and 0.101 (1.579) pg/mL for p-tau181, and −0.020 (0.279) pg/mL and −0.075 (0.215) pg/mL for t-tau. The effect sizes are negligible (< 0.20) except for moderate effect sizes for a 6-month difference in p-tau181 in the stretching group (*d*=0.43 [−0.33, 1.19]) and a 6-month difference in t-tau in the cycling group (*d*=−0.35 [−0.87, 0.17]).

**Table 2:** Observed level of plasma AD biomarkers by group at baseline, 3, and 6 months

	Baseline		3 months		6 months	
	*N*	Mean (S.D.)	*N*	Mean (S.D.)	*N*	Mean (S.D.)
Stretching						
Plasma Ab_42/40_ ratio	8	0.053 (0.013)	8	0.055 (0.011)	8	0.054 (0.010)
t-tau	8	0.872 (0.271)	8	0.865 (0.283)	8	0.851 (0.287)
p-tau181	7	2.660 (1.338)	8	2.729 (1.119)	8	3.269 (1.491)
Cycling						
Plasma Ab_42/40_ ratio	18	0.055 (0.010)	15	0.054 (0.010)	17	0.054 (0.011)
t-tau	18	0.791 (0.184)	15	0.789 (0.165)	17	0.716 (0.242)
p-tau181	15	2.505 (1.430)	15	2.561 (1.401)	16	2.606 (1.716)

**Table 3:** Effect sizes for within-group differences in plasma AD biomarkers (reference: baseline)

	Plasma Ab_42/40_ ratio		t-tau		p-tau181	
	Difference (SD^a^)	Cohen’s d^b,c^ [95% CI]	Difference (SD^a^)	Cohen’s d^b,c^ [95% CI]	Difference (SD^a^)	Cohen’s d^b,c^ [95% CI]
*Stretching*						
vs. 3 months	0.002 (0.012)	0.16 [−0.22; 0.54]	−0.007 (0.277)	−0.02 [−0.60; 0.55]	0.069 (1.234)	0.06 [−0.45; 0.56]
vs. 6 months	0.001 (0.012)	0.11 [−0.33; 0.55]	−0.020 (0.279)	−0.07 [−0.65; 0.51]	0.609 (1.417)	0.43 [−0.33; 1.19]
*Cycling*						
vs. 3 months	−0.001 (0.010)	−0.07 [−0.32; 0.19]	−0.002 (0.175)	−0.01 [−0.58; 0.55]	0.056 (1.416)	0.04 [−0.49; 0.57]
vs. 6 months	−0.001 (0.010)	−0.10 [−0.38; 0.18]	−0.075 (0.215)	−0.35 [−0.87; 0.17]	0.101 (1.579)	0.06 [−0.48; 0.60]

The sample sizes for the stretching and the cycling groups are 8 and 18, 8 and 15, and 8 and 17 at baseline, and 3 and 6 months, respective

^a^Pooled standard deviation, ^b^Cohen’s d: small (.2), medium (.5), and large (.8); ^c^bootstrap estimation (number of replications=1010)

Table 4 summarizes the results of the linear mixed model for each biomarker. No significant interactions between intervention groups and time were observed for any biomarker at the .05 level of significance with or without controlling for baseline covariates. Among the baseline covariates, age showed a significant and positive association with p-tau181, being married associated with higher plasma t-tau compared to non-married, having e2/e4 APOE genotype associated with a lower t-tau, compared to e3/e4 APOE genotype.

**Table 4:** Multilevel analysis of plasma AD biomarkers

	Plasma Ab_42/40_ ratio		t-tau		p-tau181	
	Coef.	95% CI	Coef.	95% CI	Coef.	95% CI
***Fixed effects***						
Time^a^						
3 months	0.002	[–0.001; 0.005]	–0.007	[–0.144; 0.131]	0.086	[–0.545; 0.718]
6 months	0.001	[–0.002; 0.004]	–0.020	[–0.158; 0.117]	0.627	[–0.005; 1.259]
Group^b^						
Cycling	0.003	[–0.005; 0.012]	0.019	[–0.160; 0.199]	0.322	[–0.795; 1.439]
Time×Group						
3 months×Cycling	–0.003	[–0.006; 0.001]	0.032	[–0.137; 0.201]	0.218	[–0.561; 0.996]
6 months×Cycling	–0.002	[–0.006; 0.001]	–0.040	[–0.206; 0.127]	–0.210	[–0.988; 0.567]
APOE genotype^c^						
ε2/ε3	–0.007	[–0.028; 0.013]	0.179	[–0.209; 0.567]	1.827	[–0.794; 4.448]
ε2/ε4	–0.007	[–0.018; 0.003]	–**0.234**	[–0.425; –0.043]	–0.811	[–2.079; 0.457]
ε4/ε4	–0.002	[–0.012; 0.008]	–0.046	[–0.239; 0.147]	–0.228	[–1.516; 1.060]
Age	0.000	[–0.000; 0.001]	0.007	[–0.003; 0.018]	**0.069**	[0.001; 0.138]
Female^d^	–0.006	[–0.014; 0.002]	–0.041	[–0.191; 0.110]	–0.849	[–1.846; 0.149]
White^e^	0.002	[–0.011; 0.015]	0.010	[–0.235; 0.255]	–1.617	[–3.242; 0.008]
Education in years	0.001	[–0.000; 0.002]	–0.019	[–0.043; 0.005]	–0.062	[–0.220; 0.097]
Married^f^	–0.006	[–0.017; 0.005]	**0.262**	[0.054; 0.470]	1.356	[–0.024; 2.737]
MMSE	0.000	[–0.002; 0.001]	–0.009	[–0.033; 0.016]	0.062	[–0.099; 0.224]
Constant	**0.060**	[0.045; 0.075]	0.685	[0.391; 0.978]	**3.241**	[1.336; 5.147]
***Variance components***						
Level 1: within-person	**0.000**	**[0.000; 0.000]**	**0.018**	**[0.008; 0.040]**	**0.948**	**[0.501; 1.793]**
Level 2: Initial status	**0.000**	**[0.000; 0.000]**	**0.020**	**[0.013; 0.029]**	**0.377**	**[0.247; 0.576]**
Deviance	286.07		23.81		–90.38	
AIC	–538.14		–13.62		214.75	
BIC	–498.97		25.55		252.73	
*N*	74^g^		74^g^		69^h^	

The reference groups are (a) baseline; (b) stretching group; (c) ε3/ε4; (d) male; (e) non-White; and (f) not married; bold if significant at the .05 level of significance; (g) the sample sizes for the stretching and the cycling groups are 8 and 18, 8 and 15, and 8 and 17 at baseline, and 3 and 6 months, respectively; (h) the sample sizes for the stretching and the cycling groups are 7 and 15, 8 and 15, and 8 and 16 at baseline, and 3 and 6 months, respectively

## Discussion

The increasing availability and ease of use of blood biomarkers have provided an unprecedented opportunity for monitoring treatment responses to interventions in AD. However, no studies had examined the feasibility of blood biomarkers in older adults with Alzheimer’s dementia when our FIT-AD trial was launched. This study piloted the blood component of the future Phase III RCT. Our findings show that recruitment, retention, and blood collections were highly feasible. There were potentially moderate effect sizes for 6-month within-group differences in p-tau181 in the stretching group and t-tau for the cycling group.

Aerobic exercise has been shown to modify AD biomarkers in AD-transgenic mouse models with a recent meta-analysis showing that swimming, moderate intensity, and duration of exercises exerting greater effects on Aβ [7]. The lack of blood biomarker studies in human exercise trials indicated the need for pilot studies, which was addressed by our FIT-AD blood study. Our findings showed that blood collections were well received by participants despite the need to fast for 8 h prior to blood draws. Our recruitment rate was 76.5%, which did not reach the 80% benchmark but should not be interpreted as a failure because we recruited passively by email. We knew some participants and family caregivers did not respond to emails. In addition, we made little effort to recruit by screening out 8 responders based on the staff’s judgment if they were likely to participate. Future RCTs could improve recruitment success even with minimal staff effort. For example, staff could share study information in person or over the phone or mention the study on multiple occasions during recruitment and screening. Blood collections could also be designed as an essential component of a trial.

Our retention and blood collection rates were all above 80%, achieving a retention rate of 100% at 3 months and 96.2% at 6 months. The only participant we lost was due to non-study-related death. The rate of blood collection was 88.5% at 3 months and 96.2% at 6 months. We could have increased blood collection rates at 3 months if we had been flexible with our data collection window. Future RCTs could build the following strategies into the trial design: a reasonably large data collection window like 3 weeks instead of the 1-week window we used, home-based blood collections at least for special circumstances, blood collections at a local laboratory close to a participant’s residence, and transportation assistance if blood collections must occur in a central location. Collaborating with participants and family caregivers to co-create strategies is also important to minimize barriers to blood collection.

A preliminary result from our study is the potential effects of aerobic exercise on AD pathophysiology, by a trend of a small increase in plasma p-tau181 in the cycling group. Studies examining physical activity and tau are very limited, with some showing an association between moderate physical activity and reduced CSF p-tau [37] while others reporting no association [38, 39]. A 16-week aerobic exercise RCT failed to change CSF tau in older adults with mild-to-moderate Alzheimer's dementia [31]. Our study suggested that aerobic exercise may have a moderate effect size on reducing or slowing down the increase of plasma p-tau181 at 6 months.

Prior studies suggested that biomarker dynamics differ in AD, with both CSF and plasma Aβ_42/40_ ratio starting to change before p-tau [26], p-tau levels continuing to increase as AD pathology accumulates [40], and the increases in p-tau in magnitude being considerably larger than the decrease of Aβ_42/40_ ratio [12, 26]. Greater disease-related changes in plasma p-tau181 than in plasma Aβ_42/40_ ratio are consistent with our findings on greater within-group effect sizes in plasma p-tau 181 than plasma Aβ_42/40_ ratio.

The trends observed in our study, if reproducible in future large-scale trials, would suggest the disease-modifying ability of aerobic exercise in older adults with Alzheimer's dementia. This would be ground-breaking because the degree and location of p-tau abnormality in the brain are associated with the onset and severity of cognitive symptoms [44]. Moreover, if exercise interventions could modify plasma p-tau at the dementia phase of AD, as suggested in our study, lifelong exercise habits would be more likely to be beneficial for AD prevention in the preclinical and MCI phases of AD.

There are several strengths in our study. We conducted a high-quality biomarker collection after overnight fasting and analysis using the established Simoa assays and ran biomarkers in duplicates to ensure accuracy. Our study is one of the few exercise interventions to use a longitudinal design and collect blood samples at three timepoints. The FIT-AD trial was rigorously designed and implemented. Stretching was matched to the aerobic-exercise intervention to serve as the attention control. Older adults with Alzheimer's dementia were randomized to the intervention and control groups instead of using cognitively normal older adults as the control because they differ substantially from those with Alzheimer's dementia in many important lifestyle and clinical profiles. Our study is limited by its small sample size, lack of power, inability to account for physical activity outside the study, and the inclusion of only a subset of the FIT-AD sample. The confidence intervals on the changes in the plasma biomarkers were wide and imprecise, which is attributable to our small sample size and the lack of adjustment for important covariates such as AD severity. Although participants were consecutively recruited, consented, and enrolled prior to the FIT-AD Trial randomization, it is possible that the self-selection nature may have affected the study findings. Our findings are further limited by the inability of the plasma biomarkers to determine brain region-specific changes [8].

In summary, our findings indicate the feasibility of recruiting older adults with mild-to-moderate Alzheimer’s dementia with blood biomarkers as an outcome measure. Blood biomarkers have the potential to significantly broaden AD research in historically under-represented communities in AD research. People in these communities may have difficulty getting to academic centers with imaging and/or be more hesitant to consent to imaging or CSF procedures, although lifestyle interventions may be particularly important to these communities as a potential beneficial therapy. Our study provides the design and implementation strategies to ensure high recruitment, retention, and blood collection rates and within-group effect size estimates for future exercise RCTs.

## Conclusion

Blood biomarkers are well received by participants and feasible for exercise trials with high recruitment, retention, and blood collection rates. The effect size of the within-group 6-month difference for p-tau181 is moderate. Important trial design strategies and potential effect sizes are provided to inform future trials.

## Supplementary Information

**Additional file 1: Supplement Table.** CONSORT 2010 checklist of information to include when reporting a pilot or feasibility trial.

**Additional file 2.**

## Data Availability

All data generated or analyzed during this study are included in this published [and its supplementary information files].

## Abbreviations

Aβ_42/40_: Amyloid-β_42_ amyloid-β_40_ ratio
Aβ: Amyloid-beta
AD: Alzheimer’s disease
AT(N): Amyloid-beta, hyperphosphorylated Tau, and neurodegeneration
CDR: Clinical dementia rating
CSF: Cerebrospinal fluid
CV: Coefficient of variation
EDTA: Ethylenediaminetetraacetic acid
18 F-FDG-PET: Fluorine-18 fluorodeoxyglucose positron emission tomography
HRR: Heart rate reserve
MMSE: Mini-Mental State Examination
MRI: Magnetic resonance imaging
PET: Positron emission tomography
p-tau181: Plasma tau hyperphosphorylated at threonine 181
p-tau 217: Plasma tau hyperphosphorylated at threonine 217
QC: Quality control
RCTs: Randomized controlled trials
RPE: Ratings of perceived exertion
SD: Standard deviations
t-tau: Total tau

## References

[1] Association As (2021). 2021 Alzheimer’s disease facts and figures.

[2] McCleery J, Quinn TJ (2021). Aducanumab and the certainty of evidence. Age Ageing.

[3] Livingston G, Sommerlad A, Orgeta V, Costafreda SG, Huntley J, Ames D (2017). Dementia prevention, intervention, and care. Lancet.

[4] Santos-Lozano A, Pareja-Galeano H, Sanchis-Gomar F, Quindos-Rubial M, Fiuza-Luces C, Cristi-Montero C (2016). Physical activity and Alzheimer disease: a protective association. Mayo Clin Proc.

[5] Rabin JS, Klein H, Kirn DR, Schultz AP, Yang HS, Hampton O (2019). Associations of physical activity and beta-amyloid with longitudinal cognition and neurodegeneration in clinically normal older adults. JAMA Neurol.

[6] Pisani S, Mueller C, Huntley J, Aarsland D, Kempton MJ (2021). A meta-analysis of randomised controlled trials of physical activity in people with Alzheimer’s disease and mild cognitive impairment with a comparison to donepezil. Int J Geriatr Psychiatry.

[7] Vasconcelos-Filho FSL, da Rocha Oliveira LC, de Freitas TBC, de Pontes P, Rocha ESRCD, Godinho WDN (2021). Effect of involuntary chronic physical exercise on beta-amyloid protein in experimental models of Alzheimer’s disease: systematic review and meta-analysis. Exp Gerontol.

[8] Zetterberg H, Blennow K. Moving fluid biomarkers for Alzheimer’sdisease from research tools to routineclinical diagnostics. Mol Neurodegener. 2021;16(10). https://molecularneurodegeneration.biomedcentral.com/articles/10.1186/s13024-021-00430-x. 10.1186/s13024-021-00430-x PMC7893769 33608044

[9] Simren J, Ashton NJ, Blennow K, Zetterberg H (2020). An update on fluid biomarkers for neurodegenerative diseases: recent success and challenges ahead. Curr Opin Neurobiol.

[10] Olsson B, Lautner R, Andreasson U, Ohrfelt A, Portelius E, Bjerke M (2016). CSF and blood biomarkers for the diagnosis of Alzheimer’s disease: a systematic review and meta-analysis. Lancet Neurol.

[11] Janelidze S, Stomrud E, Palmqvist S, Zetterberg H, van Westen D, Jeromin A (2016). Plasma beta-amyloid in Alzheimer’s disease and vascular disease. Sci Rep.

[12] Nakamura A, Kaneko N, Villemagne VL, Kato T, Doecke J, Dore V (2018). High performance plasma amyloid-beta biomarkers for Alzheimer’s disease. Nature.

[13] Ovod V, Ramsey KN, Mawuenyega KG, Bollinger JG, Hicks T, Schneider T (2017). Amyloid beta concentrations and stable isotope labeling kinetics of human plasma specific to central nervous system amyloidosis. Alzheimers Dement.

[14] Palmqvist S, Janelidze S, Stomrud E, Zetterberg H, Karl J, Zink K (2019). Performance of fully automated plasma assays as screening tests for alzheimer disease-related beta-amyloid status. JAMA Neurol.

[15] Schindler SE, Bollinger JG, Ovod V, Mawuenyega KG, Li Y, Gordon BA (2019). High-precision plasma beta-amyloid 42/40 predicts current and future brain amyloidosis. Neurology.

[16] Hansson O, Lehmann S, Otto M, Zetterberg H, Lewczuk P (2019). Advantages and disadvantages of the use of the CSF Amyloid beta (Abeta) 42/40 ratio in the diagnosis of Alzheimer’s disease. Alzheimers Res Ther.

[17] Verberk IMW, Slot RE, Verfaillie SCJ, Heijst H, Prins ND, van Berckel BNM (2018). Plasma amyloid as prescreener for the earliest Alzheimer pathological changes. Ann Neurol.

[18] Vergallo A, Megret L, Lista S, Cavedo E, Zetterberg H, Blennow K (2019). Plasma amyloid beta 40/42 ratio predicts cerebral amyloidosis in cognitively normal individuals at risk for Alzheimer’s disease. Alzheimers Dement.

[19] Janelidze S, Mattsson N, Palmqvist S, Smith R, Beach TG, Serrano GE (2020). Plasma P-tau181 in Alzheimer’s disease: relationship to other biomarkers, differential diagnosis, neuropathology and longitudinal progression to Alzheimer’s dementia. Nat Med.

[20] Karikari TK, Benedet AL, Ashton NJ, Lantero Rodriguez J, Snellman A, Suarez-Calvet M (2021). Diagnostic performance and prediction of clinical progression of plasma phospho-tau181 in the Alzheimer’s Disease Neuroimaging Initiative. Mol Psychiatry.

[21] Karikari TK, Pascoal TA, Ashton NJ, Janelidze S, Benedet AL, Rodriguez JL (2020). Blood phosphorylated tau 181 as a biomarker for Alzheimer’s disease: a diagnostic performance and prediction modelling study using data from four prospective cohorts. Lancet Neurol.

[22] Lantero Rodriguez J, Karikari TK, Suarez-Calvet M, Troakes C, King A, Emersic A (2020). Plasma p-tau181 accurately predicts Alzheimer’s disease pathology at least 8 years prior to post-mortem and improves the clinical characterisation of cognitive decline. Acta Neuropathol.

[23] Palmqvist S, Janelidze S, Quiroz YT, Zetterberg H, Lopera F, Stomrud E (2020). Discriminative accuracy of plasma phospho-tau217 for Alzheimer disease vs other neurodegenerative disorders. JAMA.

[24] Thijssen EH, La Joie R, Wolf A, Strom A, Wang P, Iaccarino L (2020). Diagnostic value of plasma phosphorylated tau181 in Alzheimer’s disease and frontotemporal lobar degeneration. Nat Med.

[25] Mielke MM, Hagen CE, Xu J, Chai X, Vemuri P, Lowe VJ (2018). Plasma phospho-tau181 increases with Alzheimer’s disease clinical severity and is associated with tau- and amyloid-positron emission tomography. Alzheimers Dement.

[26] Palmqvist S, Insel PS, Stomrud E, Janelidze S, Zetterberg H, Brix B (2019). Cerebrospinal fluid and plasma biomarker trajectories with increasing amyloid deposition in Alzheimer’s disease. EMBO Mol Med.

[27] O’Connor A, Karikari TK, Poole T, Ashton NJ, Lantero Rodriguez J, Khatun A, et al. Plasma phospho-tau181 in presymptomatic and symptomatic familial Alzheimer’s disease: a longitudinal cohort study. Mol Psychiatry. 2021;26:5967–76. 10.1038/s41380-020-0838-x PMC7612227 32665603

[28] Vidoni ED, Morris JK, Watts A, Perry M, Clutton J, Van Sciver A (2021). Effect of aerobic exercise on amyloid accumulation in preclinical Alzheimer’s: a 1-year randomized controlled trial. PLoS One.

[29] Baker LD, Frank LL, Foster-Schubert K, Green PS, Wilkinson CW, McTiernan A (2010). Effects of aerobic exercise on mild cognitive impairment: a controlled trial. Arch Neurol.

[30] Steen Jensen C, Portelius E, Siersma V, Hogh P, Wermuth L, Blennow K (2016). Cerebrospinal fluid amyloid beta and tau concentrations are not modulated by 16 weeks of moderate- to high-intensity physical exercise in patients with Alzheimer disease. Dement Geriatr Cogn Disord.

[31] Jensen CS, Portelius E, Hogh P, Wermuth L, Blennow K, Zetterberg H (2017). Effect of physical exercise on markers of neuronal dysfunction in cerebrospinal fluid in patients with Alzheimer’s disease. Alzheimers Dement (N Y).

[32] Frederiksen KS, Madsen K, Andersen BB, Beyer N, Garde E, Hogh P (2019). Moderate- to high-intensity exercise does not modify cortical beta-amyloid in Alzheimer’s disease. Alzheimers Dement (N Y).

[33] Yu F, Mathiason MA, Gunter JL, Jones D, Botha H, Jack C (2021). Mechanistic effects of aerobic exercise in Alzheimer’s disease: imaging findings from the pilot FIT-AD Trial. Front Aging Neurosci.

[34] Eldridge SM, Lancaster GA, Campbell MJ, Thabane L, Hopewell S, Coleman CL (2016). Defining feasibility and pilot studies in preparation for randomised controlled trials: development of a conceptual framework. PLoS One.

[35] Yu F, Bronas UG, Konety S, Nelson NW, Dysken M, Jack C (2014). Effects of aerobic exercise on cognition and hippocampal volume in Alzheimer’s disease: study protocol of a randomized controlled trial (The FIT-AD trial). Trials.

[36] Yu F, Vock DM, Zhang L, Salisbury D, Nelson NW, Chow LS (2021). Cognitive effects of aerobic exercise in Alzheimer’s disease: a pilot randomized controlled trial. J Alzheimers Dis.

[37] Law LL, Rol RN, Schultz SA, Dougherty RJ, Edwards DF, Koscik RL (2018). Moderate intensity physical activity associates with CSF biomarkers in a cohort at risk for Alzheimer’s disease. Alzheimers Dement (Amst).

[38] Frederiksen KS, Gjerum L, Waldemar G, Hasselbalch SG (2019). Physical activity as a moderator of Alzheimer pathology: a systematic review of observational studies. Curr Alzheimer Res.

[39] Reijs BLR, Vos SJB, Soininen H, Lotjonen J, Koikkalainen J, Pikkarainen M (2017). Association between later life lifestyle factors and Alzheimer’s disease biomarkers in non-demented individuals: a longitudinal descriptive cohort study. J Alzheimers Dis.

[40] Mattsson-Carlgren N, Andersson E, Janelidze S, Ossenkoppele R, Insel P, Strandberg O (2020). Abeta deposition is associated with increases in soluble and phosphorylated tau that precede a positive Tau PET in Alzheimer’s disease. Sci Adv.

[41] Li D, Thomas R, Tsai MY, Li L, Vock DM, Greimel S (2016). Vascular biomarkers to predict response to exercise in Alzheimer’s disease: the study protocol. BMJ Open.

[42] Rozga M, Bittner T, Batrla R, Karl J (2019). Preanalytical sample handling recommendations for Alzheimer's disease plasma biomarkers. Alzheimers Dement (Amst)..

[43] Kadel R, Kip KA, editors. SAS macro to compute effect size (Cohen’sd) and its confidence interval from raw survey data. Proceedings of the annual southeast SAS users group conference. 2012.

[44] Di J, Cohen LS, Corbo CP, Phillips GR, El Idrissi A, Alonso AD (2016). Abnormal tau induces cognitive impairment through two different mechanisms: synaptic dysfunction and neuronal loss. Sci Rep.

