# Supplementary material for: Feasibility and preliminary effects of exercise interventions on plasma biomarkers of Alzheimer’s disease in the FIT-AD trial: a randomized pilot study in older adults with Alzheimer’s dementia
Source: Pilot Feasibility Stud. 2022 Dec 2;8:243. doi: 10.1186/s40814-022-01200-2 (PMC9716660; doi:10.1186/s40814-022-01200-2)
Supplement: Supplementary file 2 — Additional file 2. [file 40814_2022_1200_MOESM2_ESM.pdf]

Yu et al. Trials 2014, 15:394
http://www.trialsjournal.com/content/15/1/394

TRIALS

STUDY PROTOCOL Open Access

Effects of aerobic exercise on cognition and hippocampal volume in Alzheimer's disease: study protocol of a randomized controlled trial (The FIT-AD trial)

Fang Yu[1*], Ulf G Bronas[1], Suma Konety[2], Nathaniel W Nelson[3], Maurice Dysken[4], Clifford Jack Jr[5], Jean F Wyman[1], David Vock[6] and Glenn Smith[7]

Abstract

Background: Alzheimer's disease, a global public health issue, accounts for 60 to 80% of all dementias. Alzheimer's disease primarily causes cognitive impairment and drugs have only modest short-term effects, highlighting a pressing need to develop effective interventions. Aerobic exercise holds promise for treating cognitive impairment in Alzheimer's disease through biologically sound mechanisms. Nonetheless, aerobic exercise studies in Alzheimer's disease are limited with mixed findings.

Methods/Design: This pilot randomized controlled trial will investigate the effects of a 6-month, individualized, moderate-intensity cycling intervention (20 to 50 minutes per session, 3 times a week) on cognition and hippocampal volume in community-dwelling older adults with mild-to-moderate Alzheimer's disease. The specific aims are to: 1) determine the immediate effect of the cycling intervention on cognition in Alzheimer's disease; 2) examine if the cycling intervention slows cognitive decline in Alzheimer's disease from baseline to 12 months; and 3) assess the effect of aerobic exercise on hippocampal volume over 12 months. Ninety subjects will be randomized on a 2:1 allocation ratio to cycling or attention control (low-intensity stretching) and followed for another 6 months. Allocations will be concealed to all investigators and outcome assessors will be blinded to group assignments and previous data. Cognition will be measured by the Alzheimer's disease Assessment Scale-Cognition at baseline before randomization and at 3, 6, 9, and 12 months. Hippocampal volume will be measured by magnetic resonance imaging at baseline and 6 and 12 months. The sample size of 90 will give 80% power to detect a 2.5-point difference in within-group changes in the Alzheimer's disease Assessment Scale-Cognition at 6 months for the cycling group.

Discussion: Findings from this study will address the critical gap of exercise efficacy in Alzheimer's disease and use of magnetic resonance imaging as an outcome measure in clinical trials. This study will provide a potential treatment that may increase physical function and quality of life and curb the prohibitive costs for the growing dementia population.

Trial registration: Primary registration: (NCT01954550; date of registration: 20 September 2013). Secondary registration: (NCT01954550; date of registration: 1 October 2013).

Keywords: Exercise, Alzheimer's disease, Dementia, Physical activity, Cognition, Hippocampal volume, Imaging

* Correspondence: yuxxx244@umn.edu
[1]University of Minnesota School of Nursing, 5-160 WDH 1331, 308 Harvard St SE, Minneapolis, MN 55455, USA
Full list of author information is available at the end of the article

BioMed Central

© 2014 Yu et al.; licensee BioMed Central Ltd. This is an Open Access article distributed under the terms of the Creative Commons Attribution License (http://creativecommons.org/licenses/by/4.0), which permits unrestricted use, distribution, and reproduction in any medium, provided the original work is properly credited. The Creative Commons Public Domain Dedication waiver (http://creativecommons.org/publicdomain/zero/1.0/) applies to the data made available in this article, unless otherwise stated.

Background

Dementia is a global public health issue, affecting 35.6 million people and costing $604 billion worldwide in 2010. Alzheimer's disease (AD) is the most common type of dementia and accounts for 60 to 80% of all dementias [1]. The hallmark symptoms of AD are cognitive impairment (in at least two cognitive domains) and functional decline, which can lead to behavioral and psychological symptoms of dementia (BPSD), loss of independence, poor quality of life, and premature institutionalization [2,3]. In addition, available drugs have only modest short-term effects on reducing cognitive impairment in AD [4]. Hence, there is a pressing need to develop non-pharmacological interventions for alleviating AD symptoms. To this end, aerobic exercise is promising due to its ability to increase gray-matter volumes in the hippocampus, improve cerebral blood flow, neuroplasticity and neurogenesis, increase production and function of neurotransmitters and neurotrophic growth factors, and reduce AD pathologic β-amyloid load [5-7].

Effect of aerobic exercise on cognition

At least five meta-analyses of randomized controlled trials (RCTs) have been conducted demonstrating that aerobic exercise produced mild-to-moderate cognitive gains in adults without AD and dementias [8-12]. Similar cognitive improvements were found from aerobic exercise in persons with mild cognitive impairment (MCI) and dementia [13-16]. One meta-analysis even showed that aerobic exercise improved memory to a greater degree in persons with MCI (Hedges' g = 0.237, $P = .05$) than in those without MCI (g = 0.096, $P = .143$) [12]. Together, these findings lend preliminary support to that aerobic exercise provides a therapeutic effect on cognition in AD, which is demonstrated by emerging aerobic exercise studies in AD. Cycling or walking for 5 to 12 weeks improved scores on the Mini-Mental State Examination (MMSE) from 16.3 at baseline to 19.8 post training ($P < .001$, n = 35) [17]. In another study, 15 men with AD increased scores on the Test of Attention Matrix from 35.9 to 43.0 and the MMSE from 19.4 to 21.7 from baseline to 3 months (all $P < .001$) after completing a 3-month moderate-intensity cycling intervention [18]. However, these studies did not have a control group.

Other researchers have found that aerobic exercise did not necessarily result in dramatic cognitive gains, but was potentially involved in stemming the progression of cognitive decline in AD. For instance, relative stability in cognitive status, as measured by the AD Assessment Scale-Cognition (ADAS-Cog), was observed in a sample of 11 [19] and 26 [20]) subjects who underwent moderate-intensity cycling for 6 months.

Furthermore, multicomponent interventions with aerobic exercise as a component showed differing effects. One study reported improved cognition as measured by the French Rapid Evaluation of Cognitive Function scale (n = 38) [21], but other studies demonstrated no changes on the Boston Naming Test (n = 27) [22] or the Hopkins Verbal Learning Test (n = 11) [23].

Together, the above findings indicate that aerobic exercise could either maintain or improve cognition in persons with AD, which are very positive findings given that progressively worsening cognition marks the course of AD. Nonetheless, aerobic exercise studies in AD are few and have been limited by such factors as small sample sizes, variable comparator groups, inconsistent cognitive measures, variable domains of cognition as a main outcome, and application of multicomponent interventions that masked the true effect of aerobic exercise [24]. The comparability of those findings is further limited due to the varied doses of aerobic exercise [24]. The doses rarely met the weekly 150-minute moderate-intensity level of exertion that has been recommended for older adults or the 6-month duration considered sufficient for producing cognitive gains in non-AD samples [7]. The prescribed exercise frequency ranged from 1 to 5 times a week, session duration from 20 to 40 minutes, intensity from very low to moderate, and program duration from 5 weeks to 4 years across the studies [17-21,23,25-31]. In addition, there was substantial discrepancy between prescribed and delivered exercise doses. Methods for ensuring exercise delivery are often unreported, while the retention and adherence rates ranged from 59 to 92% and 10 to 90%, respectively [19-22,25,27-31]. It is likely that a dose–response relationship between exercise and cognition exists, but remains unknown due to unclear exercise adherence and treatment fidelity.

Effect of aerobic exercise on hippocampal volume

AD neuropathology includes β-amyloid plaques and neurofibrillary tangles that cause neurodegeneration and brain atrophy that begins and is most severe in the medial temporal lobe, particularly in the hippocampus [32]. To measure those changes *in vivo*, magnetic resonance imaging (MRI) has been developed and validated as a tool for segmenting and calculating whole and regional brain volumes, indicating an annual 3 to 5% decline in hippocampal volume in AD [33-35]. While MRI has been studied for diagnosing AD and predicting the progression from mild cognitive impairment to AD, its use as an outcome measure in RCTs is just emerging [36-41]. Although no aerobic exercise studies in AD have used MRI, aerobic exercise has been found to increase hippocampal volume in nondemented individuals [42-44].

In summary, the current evidence supports the hypothesis that aerobic exercise might reduce cognitive decline in AD and limit the progression of AD neuropathology. However, previous studies often did not use a control

group and lack a rigorously designed and delivered aerobic exercise intervention. Even if aerobic exercise shows no direct benefits to AD's symptoms and progression, persons with AD will nevertheless receive the same health benefits from aerobic exercise, such as improved physical function, as any other populations.

Study aims

The objective of this paper is to report the study protocol of a pilot RCT that investigates the effects of a 6-month, individualized, moderate-intensity cycling intervention on cognition and hippocampal volume in AD by piloting all components of a future full-scale RCT. The study is funded by the National Institutes of Health's National Institute on Aging (1R01AG043392-01A1, 1 August 2013 to 30 April 2018). The specific aims and hypotheses of the study are:

Aim I: determine the immediate effect of the cycling intervention on cognition in AD

Hypothesis 1a Intervention subjects will have a smaller within-group increase in ADAS-Cog at 6 months than placebo subjects in AD drug RCTs.

Hypothesis 1b Control subjects will have the same within-group increase in ADAS-Cog at 6 months as placebo subjects in AD drug RCTs.

Aim II: examine if the cycling intervention slows cognitive decline in AD from baseline to 12 months

Hypothesis 2 Intervention subjects will show a smaller increase in ADAS-Cog over 12 months than control subjects.

Aim III: assess the effect of aerobic exercise on hippocampal volume in AD over 12 months using MRI

Hypothesis 3 Intervention subjects will have a smaller decrease in hippocampal volume over 12 months than control subjects.

Methods

Design

The planned design of this pilot RCT includes two parallel groups on a 2:1 (cycling:stretching) allocation ratio with allocation concealment and assessor blinding. The design adheres to the Standard Protocol Items Recommendations for Interventional Trials (SPIRIT) [45], the Consolidated Standards of Reporting Trials (CONSORT) elements [46], and the World Health Organization Trial Registration Data Set Version 1.2.1 guidelines (http://www.who.int/ictrp/network/trds/en/) for clinical trials protocols. Ninety community-dwelling older adults with mild-to-moderate AD will be enrolled: 60 will be randomized to the 6-month intervention (20 to 50-minute moderate-intensity cycling, 3 times a week) and 30 will be assigned to attention control (20 to 50-minute stretching exercise, 3 times a week; Figure 1). Cognition will be assessed at baseline before randomization and at 3, 6, 9, and 12 months, while hippocampal volume will be measured by MRI at baseline before randomization and 6 and 12 months. All data collectors will be blinded to the group assignments. This study is approved by the University of Minnesota's Institutional Review Board (IRB: #1306M35661). Informed consent will be obtained from all participants. In the event that participants could not consent for themselves, they will give assent while their families or friends, in the descending order of power of attorney, spouse, adult children, and significant others will provide surrogate consent.

Setting

Different aspects of the study activities will be conducted at different sites, including the university's Clinical and Translational Science Institute (CTSI), Center for Magnetic Resonance Resources, School of Nursing Laboratory of Clinical Physiology, Aging and Dementia Imaging Research laboratory at Mayo Clinic, MN, Young Men's Christian Association (YMCA) gymnasiums, and exercise rooms of retirement communities. Subject screening and data collections will occur at the CTSI. MRI scan will be conducted at the Center for Magnetic Resonance Resources, but MRI quality control and data processing will happen at the Aging and Dementia Imaging Research laboratory at Mayo Clinic. Exercise testing will be conducted at the Laboratory of Clinical Physiology. Cycling and stretching exercises will be delivered at YMCA gyms, exercise rooms of the retirement communities, or the Laboratory of Clinical Physiology, to minimize travel for the subjects. Study staff involved in exercise delivery will provide transportation for all enrolled subjects to attend all study activities using a rented university vehicle with liability coverage.

Study population

The study population is community-dwelling older adults with mild-to-moderate AD. The inclusion and exclusion criteria for the study sample are detailed in Table 1. Mild-to-moderate AD will be defined as Clinical Dementia Rating (CDR) scores of 0.5 to 2 and Mini-Mental State Examination (MMSE) scores of 15 to 26. We will proactively recruit minorities using recommended strategies, such as providing educational seminars and hiring ethnically diverse staff [47].

Sample size

The primary endpoint is the 6-month within-group change in the ADAS-Cog, compared with the mean increase of 3.2

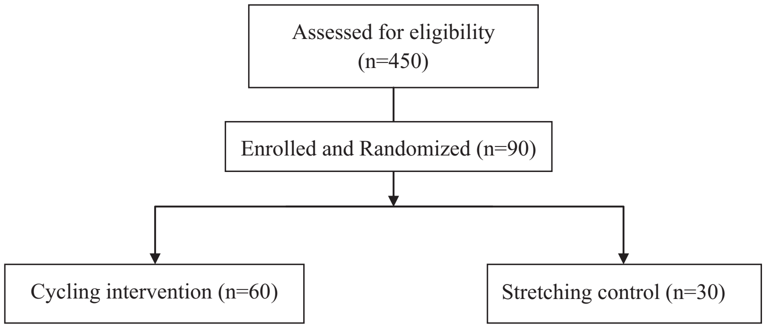

Figure 1 Subject enrollment flow diagram.

points from pooled placebo groups in AD drug RCTs with a total sample size of 534 (assuming equal numbers of mild and moderate AD subjects) [48]. Power calculations were based on a one-sample t-test (2-sided) at the 0.05 level, using the pooled standard deviation (SD) = 6.25 from the same placebo groups in AD drug RCTs [48]. Sample size was further determined by factoring in 16% attrition, which was based on the attrition rates from our previous studies [19,20], 12 to 41% attritions in AD drug RCTs [49], and 0 to 41% attritions in non-AD exercise RCTs [12]. As a result, we will enroll 90 subjects and randomize 60 to the cycling group and 30 to the control group. The power calculation is conservative as it does not account for the stratified randomized design (or equivalently assumes that there is no difference between the strata, see below).

If 50 subjects in the exercise group complete the 6-month measurement, then the study has 80% power to detect a difference of at least 2.5 points with a one-sample 2-sided t-test at the 0.05 level. That is, if cycling leads to a true mean increase in the ADAS-Cog of no more than 0.7 points, the study will have 80% power to detect a difference from the increase of 3.2 observed in placebo groups in AD drug RCTs. In our control group, if 25 complete the trial, then the expected half-width of the 95% confidence interval for the mean 6-month change in the control group is 2.4. Although there is little power to detect a difference between our controls and the placebo groups in AD drug RCTs, our trial will provide a valuable estimate of the intervention's Hawthorne effect.

Study procedure

Staff training

All staff will be adequately trained by the research team to ensure subject safety, blinding, data quality, and study-protocol adherence. Initial staff training spans three months using a variety of methods including reading, lectures, demonstration, discussions, role plays, practice rounds, and test runs with real subjects. Subsequent training of research assistants (RAs) and volunteers are conducted jointly by staff and the investigators and tailored to the skills needed for a given role. Ongoing training for staff, RAs, and volunteers is provided by the research team through weekly staff meeting, monthly research team meeting, briefing after each treatment fidelity check, and participation in new staff/RA/volunteer training.

Randomization, allocation concealment, and blinding

Randomization will allocate subjects 2:1 (cycling: stretching) within each age stratum (66 to 75, 76 to 85, and 85+), and will use randomly permutated blocks of 3 and 6 subjects (Figure 1). We do not expect equal numbers of subjects in each age stratum, but want to ensure that the

Table 1 Eligibility criteria

Inclusion criteria:	Exclusion criteria:
a) Verified diagnosis of Alzheimer's disease	a) Resting heart rate ≤50 or ≥100 beats/minute
b) Mini-Mental State Examination score 15 to 26	b) Neurologic disorders (for example, non-Alzheimer's dementia or head trauma)
c) Clinical Dementia Rating scale score 0.5 to 2	c) Psychiatric disorders (for example, bipolar, schizophrenia, or major depression)
d) Community-dwelling	d) Alcohol or chemical dependency
e) Age 66 years and older	e) Contraindications to exercise
f) English-speaking	f) If volunteered for Magnetic Resonance Imaging, abnormal imaging findings
g) Verified exercise safety	g) New symptoms or diseases (for example, chest pain, thrombosis) that have not been evaluated by the primary care provider
h) If on Alzheimer's drugs, stable on drugs more than one month	h) Abnormal findings from the symptom-limited cycle-ergometer test (for example, cardiac ischemia or arrhythmia, inability to cycle)

two randomized groups are balanced across each age stratum. Because the effect of the intervention may vary across age strata similar to varying AD prevalence across age groups [50], this design will reduce the variability in the comparisons within and between randomized groups.

Allocation is concealed from all investigators except for the statistician. Study staff will not be blinded. Enrolled subjects will be randomized after completing baseline data collection.

Recruitment, screening, and enrollment

This study recruits in a Midwestern state of the United States. We will recruit using a variety of strategies such as referrals by local Alzheimer's Association chapter, medical centers, and health care providers, study presentations, advertisement, websites, conference exhibits, and press release. Ninety subjects will be enrolled over a 5-year period using the inclusion/exclusion criteria (Table 1). Respondents to our recruitment will undergo a 4-step screening process to qualify for the non-MRI part of the study:

1) Phone screen (5 to 10 minutes) to elicit AD diagnosis and contraindications to exercise;
2) In-person interview (2 hours) at the CTSI to obtain informed consent/assent and Health Insurance Portability and Accountability Act (HIPAA) and medical record release authorization. Then, two staff will take turns to interview the subject and family caregiver separately. For example, an RA will first interview the subject using the MMSE and health history form, while the study coordinator will interview the family caregiver using the CDR. Afterwards, the RA will interview the caregiver using the health history form, while the study coordinator will interview the subject using the CDR and conduct a focused neurologic and cardiac physical exam;
3) Medical verification of AD diagnosis, exercise safety, and MRI safety with the subject's health care providers (1 to 4 weeks). Medical verification letter will be sent if the subject's CDR is 0.5 to 2 and MMSE is 15 to 26; and
4) Symptom-limited peak cycle-ergometer test (30 to 60 minutes) to rule out cardiac ischemia and serious arrhythmia and determine peak heart rate (HR) and maximal oxygen consumption (VO_{2peak}). The test is conducted either by a trained exercise technician, a study staff/RA, and a physician co-investigator, or a trained exercise technician, a study staff/RA, and an allied health professional trained in supervising exercise testing with the physician on call.

Subjects who pass all four steps will be considered 'tentatively enrolled' if they are willing to undergo MRI with MRI safety verification in step 3 above, or 'enrolled' if they refuse to participate in MRI or without MRI safety clearance in step 3 above. Tentatively enrolled subjects will be further screened for MRI eligibility using the Volunteer Safety Screening questionnaire mandated by the Center for Magnetic Resonance Imaging, and undergo baseline MRI if deemed safe. Subjects whose scans meet the following criteria will be notified to pursue medical evaluation with their providers and excluded from the study: normal pressure hydrocephalus, brain tumor, subdural hematoma, significant post traumatic encephalomalacia, one or more large hemispheric infarctions. Subjects whose scans do not meet those MRI exclusion criteria will be formally enrolled in the study.

The subject timeline is depicted in Figure 2. The actual date of enrollment of the first subject was 3 June 2014.

Data collection

Enrolled subjects will be scheduled to complete baseline data collection (about 3 hours). Subsequent data collection will occur at 3, 6, 9, and 12 months for cognition and other outcomes, which are further described under 'Variables and Their Measures' and in Figure 2. Subjects who undergo MRI at baseline will be re-evaluated for eligibility for MRI at 6 and 12 months (Figure 2). The first five data collections will be double checked by co-investigators and serve as further training for data collectors. Data collected by one assessor will be separately scored and entered into the study database by another assessor. Data collectors will not interact with enrolled subjects except for data collections.

Participation in the cycling or stretching exercise

Within two weeks of completing baseline data collection, the subjects will start their assigned exercise. Cycling or stretching exercise consists of a total of 72 sessions that will be provided 3 times a week for 6 months. The 72 sessions will be delivered over a 27-week period to account for the 3-month data-collection week (no exercise sessions) and accommodate vacation and illness make-ups. A trained exercise interventionist will lead each exercise session and supervise up to three subjects. In each session, subjects will do a 5-minute cardiac warm-up and cool-down before and after cycling and stretching exercises following the American College of Sports Medicine's guidelines [51]. Hence, the session durations will gradually lengthen from 30 minutes to 60 minutes as subjects progress from 20 to 50 minutes of cycling at moderate intensity or stretching at low intensity over time. Upon arriving in each session, the exercise interventionist will assist subject's placement of a Polar™ Wireless HR Monitor (RS400, Lake Success, NY, USA) for continuous HR monitoring. Resting HR and blood pressure will be taken. During cycling or stretching exercise, subjects will rate their Rating of Perceived Exertion (RPE) on the 6

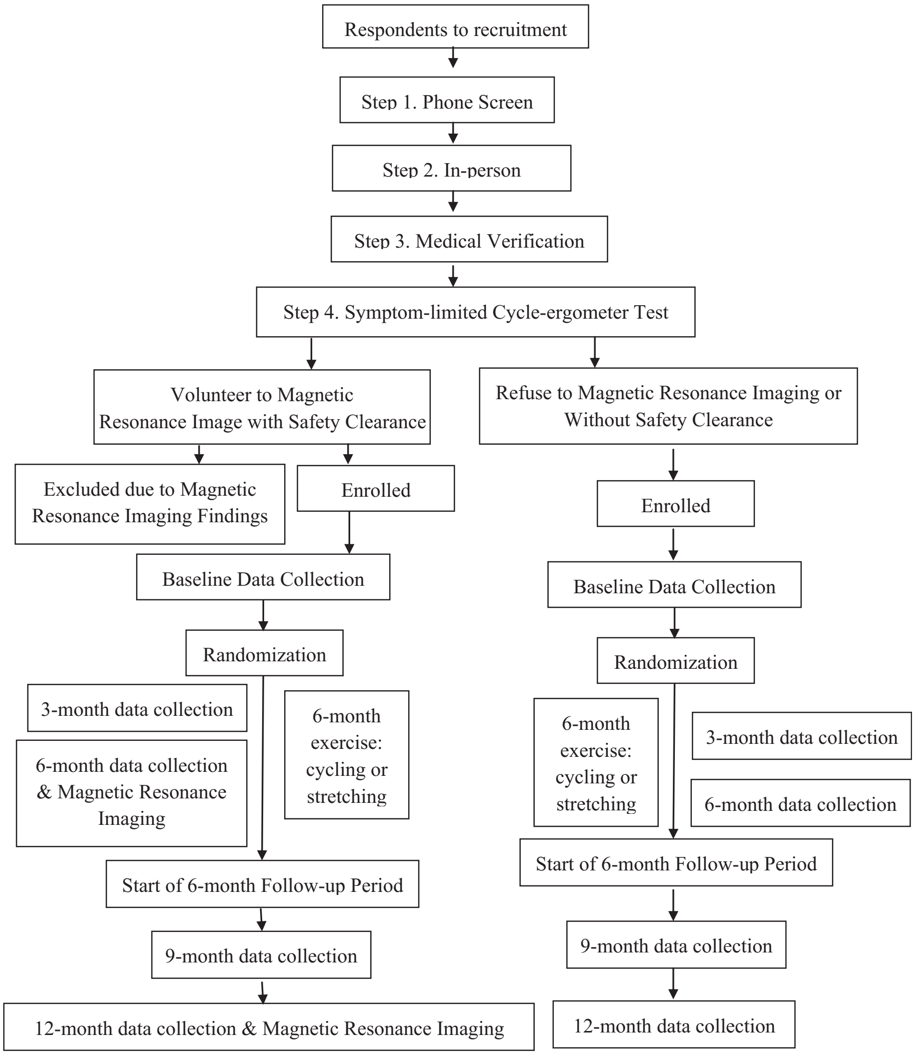

Figure 2 **Subject flow and timeline.**

to 20 Borg RPE scale every 5 minutes and corresponding HR will be documented. Blood pressure will be measured every 10 to 15 minutes, while talk test and overexertion signs and symptoms will be continuously monitored. Subjects can leave the site after their HR and blood pressure return to pre-exercise levels.

Cycling

For subjects whose HR reserve can be accurately derived from the symptom-limited peak cycle-ergometer test, we will prescribe moderate intensity exercise as 50 to 75% of HR reserve (HR reserve = peak HR-resting HR). Test-rest reliability will be established for the first ten subjects who volunteer to go through the test twice within 1 week at baseline. For subjects whose HRs cannot be used to derive HR reserve due to HR-altering drugs or irregular heart rhythm, moderate-intensity exercise will be prescribed as 9 to 14 on the 6 to 20 Borg RPE scale. The proper use of the RPE will be continuously reinforced during exercise. Therefore, for session 1, cycling intensity will be set at 50 to 55% of HR reserve or RPE 9 to 11 for 20 minutes. Cycling intensity and duration will be alternatively increased by 5% of HR reserve (or 1-point on the RPE) or 5-minute increments as tolerated up to 70 to 75% of HR reserve (or RPE 12 to 14) for 50 minutes a session over time (Table 2).

Stretching exercise

Stretching exercise will not exceed 20% of HR reserve or RPE 8 and will include primarily seated movements and static stretches. Stretching exercise will begin seated with very slow and deliberate calf raises, knee extensions, knee lifts, elbow flexion, shoulder abduction, and shoulder shrugs. Thereafter, stretches will be performed as follows: seated static calf stretch; seated anterior tibialis stretch; seated triceps stretch; seated deltoid stretch; assisted

Table 2 Cycling and sham exercise prescription and progression

Week	Cycling intensity		Sham exercise intensity		Duration (minutes)
	% of HR reserve	RPE	% of HR reserve	RPE	
1	50 to 55%	9 to 11	10 to 20%	7 to 8	20 to 30
2	50 to 55%	9 to 11	10 to 20%	7 to 8	30 to 40
3	55 to 60%	10 to 12	10 to 20%	7 to 8	30 to 40
4	55 to 60%	10 to 12	10 to 20%	7 to 8	40 to 50
5	60 to 65%	12 to 14	10 to 20%	7 to 8	40 to 50
6	60 to 65%	12 to 14	10 to 20%	7 to 8	50
7	65 to 70%	12 to 14	10 to 20%	7 to 8	50
8	65 to 70%	12 to 14	10 to 20%	7 to 8	50
9	70 to 75%	12 to 14	10 to 20%	7 to 8	50
10	70 to 75%	12 to 14	10 to 20%	7 to 8	50
11 to 24	70 to 75%	12 to 14	10 to 20%	7 to 8	50

Abbreviations: HR heart rate, *RPE* Rating of Perceived Exertion.
Durations do not include warm-up and cool-down periods (10 minutes).

seated pectoralis major stretch; seated trapezius stretch; seated neck lateral rotation (looking over shoulder slowly); seated piriformis stretch; and standing calf stretch using chair for balance. There will be a minimum 2-minute rest between stretches. The number of repetitions and durations for each stretch will be gradually increased to match the session duration of cycling (Table 2).

Treatment fidelity

This study was designed to ensure treatment fidelity based on the National Institutes of Health Behavioral Change Consortium recommendations [52]. The co-investigators such as the exercise physiologist will assess treatment fidelity on all session case reports via weekly meeting with the exercise interventionist and conduct in-person fidelity check in 2% of randomly selected exercise sessions using a standard form. The exercise interventionist will be re-trained as needed.

Exercise safety, modification, retention, adherence, and validation

Safety is the cornerstone for increasing exercise adherence and retention. As previously described, we have built-in approaches to ensure subject safety, such as the 4-step safety screening, exercise prescription based on the cycle-ergometer testing to individualize exercise prescription and progression, exercise supervision, and continuous monitoring of exercise responses (HR, blood pressure, RPE, talk ability, signs, and symptoms). Exercise adherence will be documented on the session case report forms to indicate if and what aspects of the prescription are or are not met and why. We will build in nine program-level and six staff-level strategies to improve retention and adherence (Table 3). The success of the intervention delivery will be further validated using aerobic fitness measures described below.

Variables and their measures

Primary outcome

Cognition will be measured using the ADAS-Cog as described by Rosen *et al.* [53]. The ADAS-Cog is widely used in AD drug RCTs with well-established norms [48]. It assesses orientation, memory, recall, language, and praxis

Table 3 Adherence and retention plan

**Program-level strategies:**	
1	Individualize exercise program to improve enjoyment and comfort
2	Monitor subject reactions during exercises to ensure safety
3	Exercise at sites convenient for subjects
4	Provide transportation to all study activities
5	Keep individual exercise diaries that subjects can share with families
6	Reimburse gym membership fees (will be prorated)
7	Collect data on separate days during each data-collection week (<2 hours/day)
8	Allow make-ups for vacations and illness
9	Compensate subjects (will be prorated)
**Staff-level strategies:**	
1	Know the person (for example, routine, personality, likes, dislikes, and communication patterns)
2	Guide subjects with sensitivity
3	Frequent communications with family caregivers and health care providers (for example, provide progress summary letters for upcoming doctor appointments)
4	Address unmet needs to reduce BPSD
5	Focus on achievement and progress
6	Establish rapport from day 1

Abbreviation: BPSD behavioral and psychological symptoms of dementia.

with a total score of 0 to 70 (higher score = worse function). The ADAS-Cog's interrater reliability is 0.65 to 0.99 and test-retest reliability is 0.51 to 1.0 [53]. In this pilot RCT, the ADAS-Cog will be administered at baseline and at 3, 6, 9, and 12 months.

Secondary outcome

Hippocampal volume will be determined at baseline and 6 and 12 months using the same 3 Tesla-B (3 T-B) Prisma system (Siemens, Erlangen, Germany) by the technician of the Center for Magnetic Resonance Imaging. The technician is blinded to the study aims, subject group assignments, and previous scans. The sequence includes Magnetization Prepared Rapid Gradient Echo Imaging (MPRAGE) (approximately 5 minutes), Fluid Attenuated Inversion Recovery (FLAIR) (approximately 3 minutes), and resting Blood Oxygen Level Dependent Signal (BOLD) (approximately 10 minutes). The Aging and Dementia Imaging Research Laboratory at Mayo Clinic will assist the installation and qualification of this sequence on the 3 T-B system following the same procedures established for multiple multisite studies [34]. MRI data will be transferred securely to the Mayo Clinic. The Mayo Clinic will perform quality control of each incoming image file for protocol verification, image quality, and identification of medically significant findings. The MRI will generate different measures of brain volume:

(1) Change in hippocampal volume over time (measured using longitudinal Free Surfer V5.1 [54,55]). Hippocampal volume is perhaps the most widely used morphometric measure in the AD literature [56]. Hippocampal volume rate of change is the primary volume measure;
(2) Boundary Shift Integral (BSI) measures of change in brain volume over time [57-59]; and;
(3) BSI measures of change in ventricular volume over time. BSI measures of brain and ventricle are global measures that do not require any *a priori* assumptions about where in the brain the effect of the intervention might be manifested [60,61].

Other outcomes

This pilot RCT will inform a future full-scale RCT about potential outcomes that might be more sensitive to aerobic-exercise intervention: discrete cognitive domains; exercise adherence; aerobic fitness; physical function; BPSD; and medical changes (Table 4). Discrete cognitive domains include attention, processing speed, executive function, memory, and language. Each domain will be measured using instruments established in the AD Centers' Uniform Data Set. Attention will be measured with Wechsler Digit Span. Processing speed will be measured with Wechsler Digit Symbol and Trial Making Test (TMT) Part A. Executive function will be measured using TMT Part B, Executive Interview-25 (EXIT-25) [62], Behavioral Dyscontrol Scale (BDS) [63] and Executive Clock Drawing Test (CLOX) [64]. Memory will be measured with Wechsler Memory Scale-Revised Logical Memory Story A and B Immediate and Delayed Recalls. Language will be measured with Animal and Vegetable Verbal Fluency and Boston Naming Test [65]. Exercise adherence will be calculated as: 1) the percent of the attended sessions divided by 72 for each subject; and 2) the percent of the attended sessions in which a subject meets the session prescription divided by the total attended sessions. Aerobic fitness will be measured by the symptom-limited peak cycle-ergometer [51,66] and shuttle-walk [67], and the 6-minute walk test [68], Physical function [69] will be measured by Short Physical Performance Battery (SPPB) [70], and Disability in AD (DAD) scale [71]. BPSD will be measured by the Neuropsychiatric Inventory-Caregiver (NPI-Q; [72,73]), Alzheimer's Mood Scale (AMS) [74], and Cornell Scale for Depression in Dementia (CSDD) [75]. Medical changes include changes in medical conditions, falls, medications, and health care service use and will be tracked at each exercise session. An RA will code medical changes in the previous 3 months at each data collection.

Covariates

We selected covariates that have been linked to exercise effects such as AD stage, demographics, premorbid intellect, and unsupervised physical activity (Table 4). However, the selected covariates are not meant to be all-inclusive. AD stage will be assessed using the CDR and MMSE during screening and verified during AD consensus diagnosis. Demographics include age, gender, race, and education and will be collected during screening. Premorbid intellect will be assessed at baseline using the Wechsler Test of Adult Reading (WTAR) [76]. Unsupervised physical activity will be measured by the Physical Activity Scale for the Elderly (PASE) ([77,78] and actigraph monthly for 7 days.

Data management

Data entry, coding, and storage

The principal investigator (PI) and statistician will oversee data management to ensure data accuracy and completeness. All data forms will be de-identified with paper copies double locked and entered into the username- and password-protected electronic database in Research Electronic Data Capture (REDCap). A coding book is generated in REDCap once the database is set up. REDCap is a secure web interface with data checks during data entry and uploading to ensure data quality, and is housed on secure servers operated by the University of Minnesota Academic Health Center's Information Systems. Data collection forms

Table 4 Variables assessed at specific times (S: screening, M: month)

Variables	Measures	S	M0	M3	M6	M9	M12
Primary outcome							
Global cognition	ADAS-Cog		x	x	x	x	x
Secondary outcome							
Brain volumes							
Hippocampal volume	MRI		x		x		x
Brain volume	MRI		x		x		x
Ventricle volume	MRI		x		x		x
Other outcomes							
Discreet cognitive domains							
Attention	Digit Span (Wechsler)		x	x	x	x	x
Processing speed	Digit Symbol (Wechsler)		x	x	x	x	x
	TMT Part A						
Executive function	TMT Part B		x	x	x	x	x
	EXIT-25						
	BDS Executive Clock Drawing test						
Memory	Logical Memory Story A and B		x	x	x	x	x
	Immediate Recall						
	Delayed Recall						
Language	Verbal Fluency		x	x	x	x	x
	Animal						
	Vegetable						
	Naming						
	Boston Naming Test						
Exercise adherence	Percent	Every exercise session					
Aerobic fitness	Symptom-limited peak cycle-ergometer test,	x		x	x	x	x
	Shuttle-walk test, 6-minute walk test		x	x	x	x	x
Physical function	SPPB, DAD		x	x	x	x	x
BPSD	NPI-Q, AMS, CSDD		x	x	x	x	x
Medical changes	Medical diagnoses, falls, medications, and health care service use	Monthly					
Covariates							
AD stage	CDR, MMSE	x					
Demographics	Age, education, gender, race	x					
Premorbid intellect	WTAR		x				
Unsupervised physical activity	PASE, actigraph	Monthly					

Abbreviations: AD Alzheimer's disease, *ADAS-Cog* Alzheimer's Disease Assessment Scale-Cognition, *AMS* Alzheimer's Mood Scale, *BDS* Behavioral Dyscontrol Scale, *BPSD* behavioral and psychological symptoms of dementia, *CDR* Clinical Dementia Rating, *CSDD* Cornell Scale for Depression in Dementia, *DAD* Disability in Alzheimer's Disease scale, *EXIT-25* Executive Interview-25, *MRI* magnetic resonance imaging, *MMSE* Mini-Mental State Examination, *NPI-Q* Neuropsychiatric Inventory-Caregiver, *PASE* Physical Activity Scale for the Elderly, *SPPB* Short Physical Performance Battery, *TMT* Trail Making Test, *WTAR* Wechsler Test of Adult Reading.

returned to the research office by the RAs will be reviewed by the study coordinator for completeness. Data entry will occur only after verified data completeness.

Data audits

Data audits of electronic outcome data will be conducted by the PI. If the PI identifies any discrepancies in scoring between the RA who collected data and the RA who entered data, the RAs need to consult the neuropsychologist co-investigator for correct scoring and the correct scores will be documented on paper and entered into REDCap.

Data and safety monitoring board (DSMB)

The DSMB will be comprised of three senior scientists from the University of Minnesota who are independent

of our study and will have no direct involvement with the study. The DSMB will approve the study protocol and subsequent modifications, monitor and review subject and data safety and confidentiality, subject accrual and retention, adherence to inclusion and exclusion criteria, adverse events, data quality, management, and analysis, and study progress. The DSMB will also monitor the ongoing integrity of the study and review any preliminary data analyses prepared by the statistician and annual reports. The DSMB will meet by teleconference initially, following the collection of some 3-month data, and semi-annually.

Statistical analyses

All variables will be assessed using the appropriate descriptive statistics, means and standard deviations (SD), medians, ranges, and frequencies. This approach will also allow evaluation of patterns of missing values and distribution of data. Analysis will be accomplished using SAS.

Aim 1: determine the immediate effect of the cycling intervention on cognition in Alzheimer's disease

The primary comparison for both hypotheses in Aim I will be a 2-sided test of the overall mean in a 1-way ANOVA with age stratum as the factor and the change in ADAS-Cog from baseline to 6 months as the response against the null hypothesis value of 3.2 points, which is the mean 6-month increase in ADAS-Cog in pooled placebo groups of AD drug RCTs (n = 534) [48]. Separate ANOVAs will be run for each randomization group. Because we stratified treatment assignment (by age) in the design of the study, it is important that we account for this in the analysis of the trial data in order to realize the improved power of this design. Missing values at 6 months will not be imputed by last-observation-carried-forward because this would bias the mean change toward zero. Further analysis will use an analysis of covariance (ANCOVA) to determine significant difference in ADAS-Cog between groups at 6 months after adjusting for other covariates, including baseline ADAS-Cog and age stratum as covariates. Other covariates and potential outcome variables at baseline (Table 4) will be screened for possible inclusion in the model if any are associated with ADAS-Cog at a $P < .1$ level.

Additionally, we plan to model the longitudinal trajectory of ADAS-Cog measured over the first 6 months (and then subsequently over the first 12 in Aim II). The trajectory of ADAS-Cog measures over time will first be assessed using individual subject's scatterplots and line graphs of the ADAS-Cog plotted together over time. This analysis will help to determine the appropriate function of time to model the outcome. A mixed-effects model of the ADAS-Cog measures will then be fit with fixed-effects for treatment group, (a function of) time, and their interaction and random subject-specific intercepts and slopes. The mixed-effects model will allow us to estimates of average rates of change in ADAS-Cog between the two treatment groups and assess variability in the rates of change between subjects. Mixed-effects models are advantageous because they directly accommodate correlated observations, which are expected in repeated measures, and deal readily with missing data. Additionally, we will consider longitudinal mixed-effects models with fixed-effect covariates identified in the ANCOVA and their interaction with time and treatment group. This will allow us to assess whether or not the changes in cognition and the effect of treatment group are moderated by other covariates.

Aim 2: examine if the cycling intervention slows cognitive decline in Alzheimer's disease from baseline to 12 months

Variables will be screened as in Aim I as possible covariates to be included in the longitudinal model to refine the analysis. Additionally, we will continue to document exercise participation after the intervention ends, so continued exercise may be correlated with cognition in months 9 and 12. It is speculated that cognitive decline will slow or plateau in the cycling group. Therefore, we expect change points may occur, which will be included in the model as appropriate. Mixed-effects models as described for the analysis of Aim I will be used to explore changes from baseline to 12 months to obtain the estimates needed to design a future full-scale RCT.

Aim 3: assess the effect of aerobic exercise on hippocampal volume over 12 months

Hippocampal volumes will be graphed individually to discern any recognizable patterns of change for the intervention and control subjects. The volumetric data will then be assessed from baseline to 6 and 12 months using a mixed-models analysis as in Aims 1 and 2 to determine if any indications of discernible changes in hippocampal volume were associated with the intervention. Findings will not only generate effect size estimate, but significantly inform the potential mechanism of aerobic-exercise effects in AD.

Additional analysis for other outcomes

Other outcomes, including discrete cognitive domains, exercise adherence, aerobic fitness, physical function, BPSD, and medical changes will be analyzed similarly to global cognition as described under Aim 3. Mixed-models analyses will be perform to identify any discernible changes over time.

Ethics

This study protocol is approved by the University of Minnesota IRB and DSMB. Any subsequent changes or amendments to the protocol will be submitted to IRB and DSMB for approval. Initial consent takes place during step

2 in-person screening, and subsequent consent/assent will be conducted similarly before each 3-month data collection following the same procedure during initial consent. In the event that subjects suffered research-related injury, treatment will be available, including first aid, emergency treatment and follow-up care as needed. However, care for such injuries during and after the study period will be billed in the ordinary manner to the subjects and their insurance companies.

Dissemination

Findings from the study will be disseminated through publications and presentations. Subjects and their family members will receive a copy of published main findings. Authorship will follow the established publication guidelines such as the International Committee of Medical Journal Editors. We do not intend to use professional writers. Access to the final trial data set will follow the guidelines of the National Institute on Aging of the National Institutes of Health.

Discussion

A few factors affected how we designed and operationalized this pilot RCT. First, we chose to stratify by age because we suspect that the effect of the intervention may not be consistent across ages. For example, younger subjects may be more likely to complete all the exercise regimes and do so at a higher intensity. Without stratification, there is no guarantee that the distribution of ages will be similar between the cycling and control groups and substantial sampling variability could arise among the three age strata since the sample size for this study is not large. We could by chance end up with a large percentage of 85+ in one group (cycling or control). Stratification will reduce sampling variability by ensuring a similar distribution of age in the two groups.

Second, a historical control was used for constructing hypotheses instead of utilizing the comparisons between intervention and control groups due to the small sample size and lack of power. For example, using a two-sample *t*-test, the sample size of 90 gives us 50% power to detect a group difference in the change in ADAS-Cog scores from baseline to 6 months when the true difference between the two groups is 3.5 points. We will have 80% power to detect a difference when the true difference is 5 points.

Third, we had removed an initial plan of conducting consensus diagnosis of AD using subjects' medical records. Obtaining medical records had been very difficult and time-consuming and created a bottleneck for subject screening. Providers have different rules about releasing medical records and subjects often have multiple records in different health systems, resulting in substantial delay for us to receive a subject's medical records. In addition, existing medical records rarely contain the needed data for a consensus diagnosis.

In summary, this pilot RCT will address the critical gap in aerobic exercise efficacy in AD by testing a well-characterized cycling intervention and using MRI to assess the biological mechanisms of action of aerobic exercise. This study will provide a potential treatment that may increase physical function and quality of life and curb the prohibitive costs for the growing AD population, as well as reduce caregiver burden. Our study could be easily translated into an evidence-based guideline that both professionals and the lay public can use to engage older adults with AD in aerobic exercise. If results are not as expected, the study will provide important foundations to inform future research.

Trial status

The status of the trial at the time of manuscript submission is open for enrollment and we expect enrollment accrual to complete in 2018.

Abbreviations

AD: Alzheimer's disease; ADAS-Cog: Alzheimer's Disease Assessment Scale-Cognition; AMS: Alzheimer's Mood Scale; ANCOVA: analysis of covariance; BDS: Behavioral Dyscontrol Scale; BOLD: Blood Oxygen Level Dependent Signal; BPSD: behavioral and psychological symptoms of dementia; BSI: Boundary Shift Integral; CDR: Clinical Dementia Rating; CONSORT: Consolidated Standards of Reporting Trials; CSDD: Cornell Scale for Depression in Dementia; CTSI: Clinical and Translational Science Institute; DAD: Disability in Alzheimer's Disease scale; DSMB: Data Safety and Monitoring Board; EXIT-25: Executive Interview-25; FLAIR: Fluid Attenuated Inversion Recovery; HIPAA: Health Insurance Portability and Accountability Act; HR: heart rate; IRB: Institutional Review Board; MMSE: Mini-Mental State Examination; MPRAGE: Magnetization Prepared Rapid Gradient Echo Imaging; MRI: magnetic resonance imaging; NPI-Q: Neuropsychiatric Inventory-Caregiver; PASE: Physical Activity Scale for the Elderly; PI: principal investigator; RA: research assistant; RCTs: randomized controlled trials; REDCap: Research Electronic Data Capture; RPE: Rating of Perceived Exertion; SPIRIT: Standard Protocol Items Recommendations for Interventional Trials; SPPB: Short Physical Performance Battery; TMT: Trail Making Test; VO_{2peak}: maximal oxygen consumption; WTAR: Wechsler Test of Adult Reading.

Competing interests

The authors declare that they have no competing interests.

Authors' contributions

FY designed and supervises all aspects of the study implementation, and drafted the manuscript. UGB designed and supervises all aspects of the study related to exercise, and contributed to the draft of the manuscript. SK contributed to the design of the study, supervises cycle-ergometer testing, and contributed to the draft of the manuscript. NWN contributed to the design of the study, supervises all cognitive data collections and interpretations, and contributed to the draft of the manuscript. MD contributed to the design of the study and contributed to subject screening and manuscript drafting. CJ Jr. designed all aspects of the study related to the MRI, supervises MRI data collections, and contributed to the draft of the manuscript. JFW contributed to the design of the study, provides feedback to the implementation of the study, and contributed to the draft of the manuscript. DV created and supervises randomization and drafted the manuscript. GS helped design cognitive outcome measures, provides feedback to cognitive data collection, and contributed to the draft of the manuscript. All authors read and approved the final manuscript.

Acknowledgment

The research study reported in this publication was supported by the National Institute on Aging of the National Institutes of Health Award Number 1R01AG043392-01A1. The CTSI and Center for Magnetic Resonance Resources were supported by the National Institutes of Health National Center for Advancing Translational Sciences of the National Institutes of Health Award Number UL1TR000114 and the National Institute of Biomedical Imaging and Bioengineering Award Number P41 EB1058941, respectively. The content is solely the responsibility of the authors and does not necessarily represent the official views of the National Institutes of Health.

Author details

[1]University of Minnesota School of Nursing, 5-160 WDH 1331, 308 Harvard St SE, Minneapolis, MN 55455, USA. [2]Cardiopulmonary Rehabilitation Services, University of Minnesota Medical School, Minneapolis, MN, USA. [3]University of St. Thomas, St Paul, MN, USA. [4]Minneapolis VA Health Care System, St Paul, MN, USA. [5]Department of Radiology, Mayo Clinic, Rochester, MN, USA. [6]University of Minnesota Division of Biostatistics, Minneapolis, MN, USA. [7]Departments of Psychiatry and Psychology, Mayo Clinic, Rochester, MN, USA.

Received: 16 July 2014 Accepted: 25 September 2014
Published: 11 October 2014

References

1. World Health Organization: **Dementia: a public health priority.** 2012, Retrieved from [http://whqlibdoc.who.int/publications/2012/9789241564458_eng.pdf]
2. American Psychiatric Association: *Diagnostic and Statistical Manual of Mental Disorders (4th ed.) text revision (DSM-IV-TRTM)*. Washington, DC: American Psychiatric Association; 2000.
3. Yu F, Kolanowski A, Strumpf N, Eslinger P: **Improving cognition and function through exercise intervention in Alzheimer's disease.** *J Nurs Scholarsh* 2006, **38**(4):358–365.
4. Birks J: **Cholinesterase inhibitors for Alzheimer's disease.** *Cochrane Database Syst Rev* 2006, 1. Art. No.:CD005593. doi:10.1002/14651858.CD005593.
5. Adlard AP, Perreau VM, Pop V, Cotman CW: **Voluntary exercise decreases amyloid load in a transgenic model of Alzheimer's disease.** *J of Neuroscience* 2004, **25:**4217–4221.
6. Cotman CW, Berchtold NC: **Physical activity and the maintenance of cognition: learning from animal models.** *Alzheimers Dement* 2007, **3**(Suppl 2):30–37.
7. Erickson KI, Kramer AF: **Aerobic exercise effects on cognitive and neural plasticity in older adults.** *Br J Sports Med* 2009, **43**(1):22–24.
8. Angevaren M, Aufdemkampe G, Verhaar HJ, Aleman A, Vanhees L: **Physical activity and enhanced fitness to improve cognitive function in older people without known cognitive impairment.** *Cochrane Database Syst Rev* 2008. 005381-doi:10.1002/14651858.CD005381.pub3.
9. Colcombe S, Kramer AF: **Fitness effects on the cognitive function of older adults: a meta-analytic study.** *Psychol Sci* 2003, **14**(2):125–130.
10. Etnier J, Salazar W, Landers D, Petruzzello S, Han M, Nowell P: **The influence of physical fitness and exercise upon cognitive functioning: a meta-analysis.** *J Sport and Exerc Psychol* 1997, **19**(3):249–277.
11. Etnier JL, Nowell PM, Landers DM, Sibley BA: **A meta-regression to examine the relationship between aerobic fitness and cognitive performance.** *Brain Res Rev* 2006, **52**(1):119–130.
12. Smith P, Blumenthal J, Hoffman B, Cooper H, Strauman T, Welsh-Bohmer K, Sherwood A: **Aerobic exercise and neurocognitive performance: a meta-analytic review of randomized controlled trials.** *Psychosom Med* 2010, **72**(3):239–252.
13. Baker LD, Frank LL, Foster-Schubert K, Green PS, Wilkinson CW, McTiernan A, Craft S: **Effects of aerobic exercise on mild cognitive impairment: a controlled trial.** *Arch Neurol* 2010, **67**(1):71–79.
14. Heyn P, Abreu BC, Ottenbacher KJ: **The effects of exercise training on elderly persons with cognitive impairment and dementia: a meta-analysis.** *Arch Phys Med Rehabil* 2004, **85**(10):1694–1704.
15. Scherder E, Van Paasschen J, Deijen J, Van Der Knokke S, Orlebeke J, Burgers I, Sergeant J: **Physical activity and executive functions in the elderly with mild cognitive impairment.** *Aging Mental Health* 2005, **9**(3):272–280.
16. Williams P, Lord SR: **Effects of group exercise on cognitive functioning and mood in older women.** *Australian New Zealand J Public Health* 1997, **21**(1):45–52.
17. Rolland Y, Rival L, Pillard F, Lafont C, Rivére D, Albaréde J, Vellas B: **Feasibility of regular physical exercise for patients with moderate to severe Alzheimer disease.** *J Nutr Health Aging* 2000, **4**(2):109–113.
18. Palleschi L, Vetta F, De Gennaro E, Idone G, Scottosanti G, Gianni W, Marigliano V: **Effect of aerobic training on the cognitive performance of elderly patients with senile dementia of Alzheimer type.** *Arch Gerontol Geriatrics* 1996, **5:**47–50.
19. Yu F, Nelson NW, Savik K, Wyman JF, Dyskin M, Bronas UG: **Affecting cognition and quality of life via aerobic exercise in Alzheimer's disease.** *West J Nurs Res* 2013, **35**(1):24–38.
20. Yu F, Thomas W, Nelson NW, Bronas UG, Dysken M, Wyman J: **Impact of a 6-month aerobic exercise on Alzheimer's symptoms.** *J Appl Gerontol* 2013, in press.
21. Kemoun G, Thibaud M, Roumagne N, Carette P, Albinet C, Toussaint L, Dugue B: **Effects of a physical training programme on cognitive function and walking efficiency in elderly persons with dementia.** *Dementia Geriatric Cogn Disord* 2010, **29**(2):109–114.
22. Steinberg M, Leoutsakos JS, Podewils LJ, Lyketsos C: **Evaluation of a home-based exercise program in the treatment of Alzheimer's disease: the maximizing independence in dementia (MIND) study.** *Int J Geriatric Psychiatry* 2009, **24**(7):680–685.
23. Arkin SM: **Alzheimer rehabilitation by students: Interventions and outcomes.** *Neuropsychological Rehabilitation* 2001, **11**(3–4):273–317.
24. Yu F: **Guiding research and practice: a conceptual model for aerobic exercise training in Alzheimer's disease.** *Am J Alzheimers Dis Other Dementias* 2011, **26**(3):184–194.
25. Arkin SM: **Student-led exercise sessions yield significant fitness gains for Alzheimer's patients.** *Am J Alzheimers Disease Other Dementias* 2003, **18**(3):159–170.
26. McCurry SM, Pike KC, Logsdon RG, Vitiello MV, Larson EB, Teri L: **Predictors of short- and long-term adherence to a daily walking program in persons with Alzheimer's disease.** *A J Alzheimers Dis Other Dementias* 2010, **25**(6):505–512.
27. Rolland Y, Pillard F, Klapouszczak A, Reynish E, Thomas D, Andrieu S, Vellas B: **Exercise program for nursing home residents with Alzheimer's disease: a 1-year randomized, controlled trial.** *J Am Geriatr Soc* 2007, **55**(2):158–165.
28. Tappen RM, Roach KE, Applegate EB, Stowell P: **Effect of a combined walking and conversation intervention on functional mobility of nursing home residents with Alzheimer disease.** *Alzheimer Dis Assoc Disord* 2000, **14**(4):196–201.
29. Teri L, Gibbons LE, McCurry SM, Logsdon RG, Buchner DM, Barlow WE, Larson EB: **Exercise plus behavioral management in patients with Alzheimer disease: a randomized controlled trial.** *J Am Med Assoc* 2003, **290**(15):2015–2022.
30. Williams CL, Tappen RM: **Effect of exercise on mood in nursing home residents with Alzheimer's disease.** *Am J Alzheimers Dis Other Dementias* 2007, **22**(5):389–397.
31. Williams CL, Tappen RM: **Exercise training for depressed older adults with Alzheimer's disease.** *Aging Mental Health* 2008, **12**(1):72–80.
32. Braak H, Braak E: **Neuropathological staging of Alzheimer-related changes.** *Acta Neuropathol* 1991, **82:**239–259.
33. Jack CRJ, Petersen RC, Xu Y, O'Brien PC, Smith GE, Ivnik RJ, Kokmen E: **Rate of medial temporal lobe atrophy in typical aging and Alzheimer's disease.** *Neurology* 1998, **51**(4):993–999.
34. Jack CRJ, Petersen RC, Grundman M, Jin S, Gamst A, Ward CP, Members of the Alzheimer's Disease Cooperative Study (ADCS): **Longitudinal MRI findings from the vitamin E and donepezil treatment study for MCI.** *Neurobiol Aging* 2008, **29**(9):1285–1295.
35. Mungas D, Harvey D, Reed BR, Jagust WJ, DeCarli C, Beckett L, Chui HC: **Longitudinal volumetric MRI change and rate of cognitive decline.** *Neurology* 2005, **65**(4):565–571.
36. Fox NC, Kennedy J: **Structural imaging markers for therapeutic trials in Alzheimer's disease.** *J Nutr Health Aging* 2009, **13**(4):350–352.
37. Frisoni GB, Delacourte A: **Neuroimaging outcomes in clinical trials in Alzheimer's disease.** *J Nutr Health Aging* 2009, **13**(3):209–212.
38. Hashimoto M, Kazui H, Matsumoto K, Nakano Y, Yasuda M, Mori E: **Does donepezil treatment slow the progression of hippocampal atrophy in patients with Alzheimer's disease?** *Am J Psychiatr* 2005, **162**(4):676–682.
39. Jack CRJ, Bernstein MA, Fox NC, Thompson P, Alexander G, Harvey D, Weiner MW: **The Alzheimer's disease neuroimaging initiative (ADNI): MRI methods.** *J Magn Reson Imaging* 2008, **27**(4):685–691.
40. Krishnan KR, Charles HC, Doraiswamy PM, Mintzer J, Weisler R, Yu X, Rogers S: **Randomized, placebo-controlled trial of the effects of donepezil on**

neuronal markers and hippocampal volumes in Alzheimer's disease. *Am J Psychiatr* 2003, **160**(11):2003–2011.
41. Weiner MW, Sadowsky C, Saxton J, Hofbauer RK, Graham SM, Yu SY, Perhach JL: **Magnetic resonance imaging and neuropsychological results from a trial of memantine in Alzheimer's disease.** *Alzheimers Dement* 2011, **7**(4):425–435.
42. Erickson KI, Prakash RS, Voss MW, Chaddock L, Hu L, Morris KS, Kramer AF: **Aerobic fitness is associated with hippocampal volume in elderly humans.** *Hippocampus* 2009, **19**(10):1030–1039.
43. Erickson KI, Voss MW, Prakash RS, Basak C, Szabo A, Chaddock L, Kramer AF: **Exercise training increases size of hippocampus and improves memory.** *Proc Natl Acad Sci U S A* 2011, **108**(7):3017–3022.
44. Larson EB, Wang L: **Exercise, aging, and Alzheimer disease.** *Alzheimer Dis Assoc Disord* 2004, **18**(2):54–56.
45. Chan AW, Tetzlaff JM, Altman DG, Laupacis A, Gotzsche PC, Krleza-Jeric K, Moher D: **SPIRIT 2013 statement: defining standard protocol items for clinical trials.** *Ann Intern Med* 2013, **158**(3):200–207.
46. Schulz KF, Altman DG, Moher D: **CONSORT 2010 statement: updated guidelines for reporting parallel group randomised trials.** *Ann Intern Med* 2010, **4**:291–292.
47. Welsh KA, Ballard E, Nash F, Raiford K, Harrell L: **Issues affecting minority participation in research studies of Alzheimer disease.** *Alzheimer Dis Assoc Disord* 1994, **8**(Suppl 4):38–48.
48. Doraiswamy PM, Kaiser L, Bieber F, Garman RL: **The Alzheimer's disease assessment scale: evaluation of psychometric properties and patterns of cognitive decline in multicenter clinical trials of mild to moderate Alzheimer's disease.** *Alzheimer Dis Assoc Disord* 2001, **15**(4):174–183.
49. Grill JD, Karlawish J: **Addressing the challenges to successful recruitment and retention in Alzheimer's disease clinical trials.** *Alzheimers Res Ther* 2010, **2**:34.
50. Alzheimer's Association: **Alzheimer's disease facts and figures.** 2013, [http://www.who.int/ictrp/network/trds/en/]
51. American College of Sports Medicine: *ACSM's Guidelines for Exercise Testing and Prescription (9th ed.).* Baltimore, MD: Lippincott Williams and Wilkins; 2013.
52. Bellg AJ, Borrelli B, Resnick B, Hecht J, Minicucci DS, Ory M, Treatment Fidelity Workgroup of the NIH Behavior Change Consortium: **Enhancing treatment fidelity in health behavior change studies: best practices and recommendations from the NIH behavior change consortium.** *Health Psychol* 2004, **23**(5):443–451.
53. Rosen WG, Mohs RC, Davis KL: **A new rating scale for Alzheimer's disease.** *Am J Psychiatr* 1984, **141**(11):1356–1364.
54. Dale AM, Fischl B, Sereno MI: **Cortical surface-based analysis. I. segmentation and surface reconstruction.** *NeuroImage* 1999, **9**(2):179–194.
55. Fischl B, Dale AM: **Measuring the thickness of the human cerebral cortex from magnetic resonance images.** *Proc Natl Acad Sci U S A* 2000, **97**(20):11050–11055.
56. Jack CRJ, Barkhof F, Bernstein MA, Cantillon M, Cole PE, Decarli C, Foster NL: **Steps to standardization and validation of hippocampal volumetry as a biomarker in clinical trials and diagnostic criterion for Alzheimer's disease.** *Alzheimers Dement* 2011, **7**(4):474–485.
57. Fox NC, Warrington EK, Rossor MN: **Serial magnetic resonance imaging of cerebral atrophy in preclinical Alzheimer's disease.** *Lancet* 1999, **353**(9170):2125.
58. Freeborough PA, Fox NC: **The boundary shift integral: an accurate and robust measure of cerebral volume changes from registered repeat MRI.** *IEEE Trans Med Imaging* 1997, **16**(5):623–629.
59. Gunter JL, Shiung MM, Manduca A, Jack CR: **Methodological considerations for measuring rates of brain atrophy.** *J Magn Reson Imaging* 2003, **18**(1):16–24.
60. Jack CRJ, Shiung MM, Gunter JL, O'Brien PC, Weigand SD, Knopman DS, Petersen RC: **Comparison of different MRI brain atrophy rate measures with clinical disease progression in AD.** *Neurology* 2004, **62**(4):591–600.
61. Jack CRJ, Shiung MM, Weigand SD, O'Brien PC, Gunter JL, Boeve BF, Petersen RC: **Brain atrophy rates predict subsequent clinical conversion in normal elderly and amnestic MCI.** *Neurology* 2005, **65**(8):1227–1231.
62. Royall DR, Mahurin RK, Gray KF: **Bedside assessment of executive cognitive impairment: the executive interview.** *J Am Geriatr Soc* 1992, **40**(12):1221–1226.
63. Grigsby J, Kaye K, Robbins LJ: **Reliabilities, norms and factor structure of the behavioral dyscontrol scale.** *Percept Motor Skills* 1992, **74**(3):883–892.
64. Royall DR, Cordes JA, Polk M: **CLOX: an executive clock drawing task.** *J Neurol Neurosurg Psychiatry* 1998, **64**(5):588–594.
65. Weintraub S, Salmon D, Mercaldo N, Ferris S, Graff-Radford NR, Chui H, Morris JC: **The Alzheimer's disease centers' uniform data set (UDS): the neuropsychologic test battery.** *Alzheimer Dis Assoc Disord* 2009, **23**(2):91–101.
66. Burns JM, Mayo MS, Anderson HS, Smith HJ, Donnelly JE: **Cardiorespiratory fitness in early-stage Alzheimer disease.** *Alzheimer Dis Assoc Disord* 2008, **22**(1):39–46.
67. Singh SJ, Morgan MD, Scott S, Walters D, Hardman AE: **Development of a shuttle walking test of disability in patients with chronic airways obstruction.** *Thorax* 1992, **47**(12):1019–1024.
68. Solway S, Brooks D, Lacasse Y, Thomas S: **A qualitative systematic overview of the measurement properties of functional walk tests used in the cardiorespiratory domain.** *Chest* 2001, **119**(1):256–270.
69. McAuley E, Morris KS, Doerksen SE, Motl RW, Liang H, White SM, Rosengren K: **Effects of change in physical activity on physical function limitations in older women: Mediating roles of physical function performance and self-efficacy.** *J Am Geriatr Soc* 2007, **55**(12):1967–1973.
70. Guralnik JM, Simonsick EM, Ferrucci L, Glynn RJ, Berkman LF, Blazer DG, Wallace RB: **A short physical performance battery assessing lower extremity function: association with self-reported disability and prediction of mortality and nursing home admission.** *J Gerontol* 1994, **49**(2):M85–M94.
71. Gelinas I, Gauthier L, McIntyre M, Gauthier S: **Development of a functional measure for persons with Alzheimer's disease: the disability assessment for dementia.** *Am J Occup Ther* 1999, **53**(5):471–481.
72. Cummings J: **The neuropsychiatric inventory: assessing psychopathology in dementia patients.** *Neurology* 1997, **48**(Suppl 5):10–16.
73. Kaufer DI, Cummings JL, Ketchel P, Smith V, MacMillan A, Shelley T, DeKosky ST: **Validation of the NPI-Q, a brief clinical form of the neuropsychiatric inventory.** *J Neuropsychiatr Clin Neurosci* 2000, **12**(2):233–239.
74. Tappen RM, Williams CL: **Development and testing of the Alzheimer's disease and related dementias mood scale.** *Nurs Res* 2008, **57**(6):426–435.
75. Alexopoulos GS, Abrams RC, Young RC, Shamoian CA: **Cornell scale for depression in dementia.** *Biol Psychiatry* 1988, **23**(3):271–284.
76. The Psychological Corporation: *Wechsler Test of Adult Reading.* New York: The Psychological Corporation; 2001.
77. Washburn R, Smith K, Jette A, Janney CA: **The physical activity scale for the elderly (PASE): development and evaluation.** *J Clin Epidemiol* 1993, **46**(2):153–162.
78. Washburn R, McAuley E, Katula J, Mihalko S, Boileau R: **The physical activity scale for the elderly (PASE): evidence for validity.** *J Clin Epidemiol* 1999, **52**:643–651.

doi:10.1186/1745-6215-15-394
Cite this article as: Yu *et al.*: Effects of aerobic exercise on cognition and hippocampal volume in Alzheimer's disease: study protocol of a randomized controlled trial (The FIT-AD trial). *Trials* 2014 15:394.

Submit your next manuscript to BioMed Central and take full advantage of:

- **Convenient online submission**
- **Thorough peer review**
- **No space constraints or color figure charges**
- **Immediate publication on acceptance**
- **Inclusion in PubMed, CAS, Scopus and Google Scholar**
- **Research which is freely available for redistribution**

Submit your manuscript at
www.biomedcentral.com/submit

BioMed Central
